# Endoplasmic Reticulum Stress in Cancer

**DOI:** 10.1002/mco2.70263

**Published:** 2025-06-19

**Authors:** Ruixin Zhou, Wenlong Wang, Baizhao Li, Zhu Li, Juan Huang, Xinying Li

**Affiliations:** ^1^ Department of General Surgery, Xiangya Hospital Central South University Changsha Hunan China; ^2^ National Clinical Research Center for Geriatric Disorders, Xiangya Hospital Central South University Changsha Hunan Province China; ^3^ Department of Breast Surgery, Xiangya Hospital Central South University Changsha Hunan China; ^4^ Clinical Research Center for Breast Cancer in Hunan Province Changsha Hunan China; ^5^ Molecular Bioengineering at Imperial College London London UK

**Keywords:** endoplasmic reticulum (ER), unfolded protein response (UPR), tumor microenvironment (TME), proliferation, metastasis

## Abstract

Persistent and intense endoplasmic reticulum (ER) stress is widely acknowledged as a hallmark of tumorigenesis. To restore ER homeostasis, cells activate the unfolded protein response (UPR), which is aberrantly regulated in cancer cells. This review provides an in‐depth analysis of the mechanisms through which the UPR facilitates tumor progression. The UPR is activated by ER stress sensors such as inositol‐requiring enzyme 1 (IRE1α), protein kinase R‐like ER‐resident kinase (PERK), and activating transcription factor 6 (ATF6). These sensors regulate cancer cell proliferation, immune evasion, metastasis, and drug resistance. We summarize the crosstalk between the UPR and multiple signaling pathways, including mTOR, MAPK, and NF‐κB, which collectively promote tumor growth and metastasis. Additionally, we discuss the role of the UPR in modulating the tumor microenvironment to support angiogenesis and immune evasion. We also provide an overview of pharmacological agents targeting specific UPR pathways, such as GRP78 inhibitors, IRE1α inhibitors, PERK inhibitors, and ATF6 inhibitors, with the aim of developing more effective cancer therapies. This comprehensive review highlights the potential of targeting the UPR as a novel strategy for cancer treatment and underscores the need for further research to elucidate the complex interactions between the UPR and cancer progression.

## Graphical abstract

Persistent ER stress in tumors activates UPR sensors (IRE1α, PERK, ATF6), driving cancer proliferation, metastasis, immune evasion, and drug resistance. This review explores UPR‐mediated signaling crosstalk, angiogenesis, and therapeutic targeting to develop safer anticancer strategies.

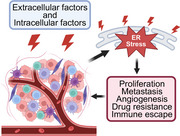

## 1 Introduction

The endoplasmic reticulum (ER), the largest cell organelle, plays a crucial role in various physiological activities, including protein transportation and folding, lipid and steroid synthesis, carbohydrate metabolism, and calcium storage [1]. Approximately 1/3 of cellular proteins are targeted to the ER, where they undergo proper folding, modification, and assembly into multiprotein complexes. These proteins are subsequently carried downstream along the secretory pathway [2]. Under normal physiological settings, protein folding and modification within the ER are highly controlled processes. However, diverse internal and external stimuli can disrupt protein homeostasis, accumulating misfolded or unfolded proteins, which are known as ER stress. When this accumulation surpasses a critical threshold, the unfolded protein response (UPR) is initiated to alleviate ER stress [3]. Extended and severe ER stress may transform the UPR into a terminal UPR [4], an alternate signaling pathway that accelerates the start and progression of numerous diseases, such as malignant cancers [5], diabetes [6], autoimmune disorders [7], and hypertension [8].

Cancer is an essential factor leading to premature noncommunicable deaths worldwide [9]. From 2018 to 2022, new cancer cases increased yearly [10, 11]. In 2022, there were 19.96 million new cancer cases and 9.74 million deaths worldwide [11]. Cancer has become a significant global public health issue [12]. Despite extensive research, the effective mechanism of cancer treatment is not yet fully understood. The crazy proliferation of cancer is the result of its escape from programmed cell death (PD), of which apoptosis is the primary way [13]. Faced with a harsh tumor microenvironment (TME), a variety of cancer cells (including breast cancer [14], liver cancer [15], pancreatic cancer [16], and lung cancer [17]) have been shown to activate high levels of UPR. On the one hand, UPR promotes the ability of cancer cells to resist stress, enhances their proliferation and metastatic potential, promotes angiogenesis, prevents immune system attacks, and induces drug resistance [18]. On the other hand, sustained high levels of UPR in some cells in the cancer cell population ultimately lead to apoptosis [19], thereby regulating the cancer microenvironment to make it more suitable for cancer growth [20]. It is worth noting that immune cells within the TME also experience severe ER stress. UPR activation in these immune cells leads to a suppressive immune microenvironment, converts anticancer responses into procancer effects, and promotes cancer immune editing and escape [21]. Increased UPR activity in the TME distinguishes cancer cells from normal cells, allowing targeted modulation of the UPR signaling pathway to treat malignancies.

In this review, we summarized the specific pathways by which UPR activates the IRE1α axis, PERK axis, and ATF6 axis under ER stress. We summarized the UPR‐promoted tumor proliferation, invasion, migration, and angiogenesis. Meanwhile, focusing on UPR promoting cancer immune escape, we discussed the mechanisms by which UPR inhibits anticancer immunity and reshapes the immunosuppressive TME through cancer cells, anticancer immune cells (NK cells, DCs, and CD8^+^ T cells), and immunosuppressive cells (M2 macrophages and myeloid‐derived suppressor cells [MDSCs]). In addition, we summarized the UPR inhibitors that have been discovered and elaborated on the pathways by which they exert their effects. Finally, we discussed the contradictions in the current research on UPR and cancer immune escape, raised questions about the development of drugs targeting UPR, and looked forward to the future development of UPR inhibitors for clinical treatment of cancers.

## 2 Mechanisms of ER Stress in Cancer

**FIGURE 1:**
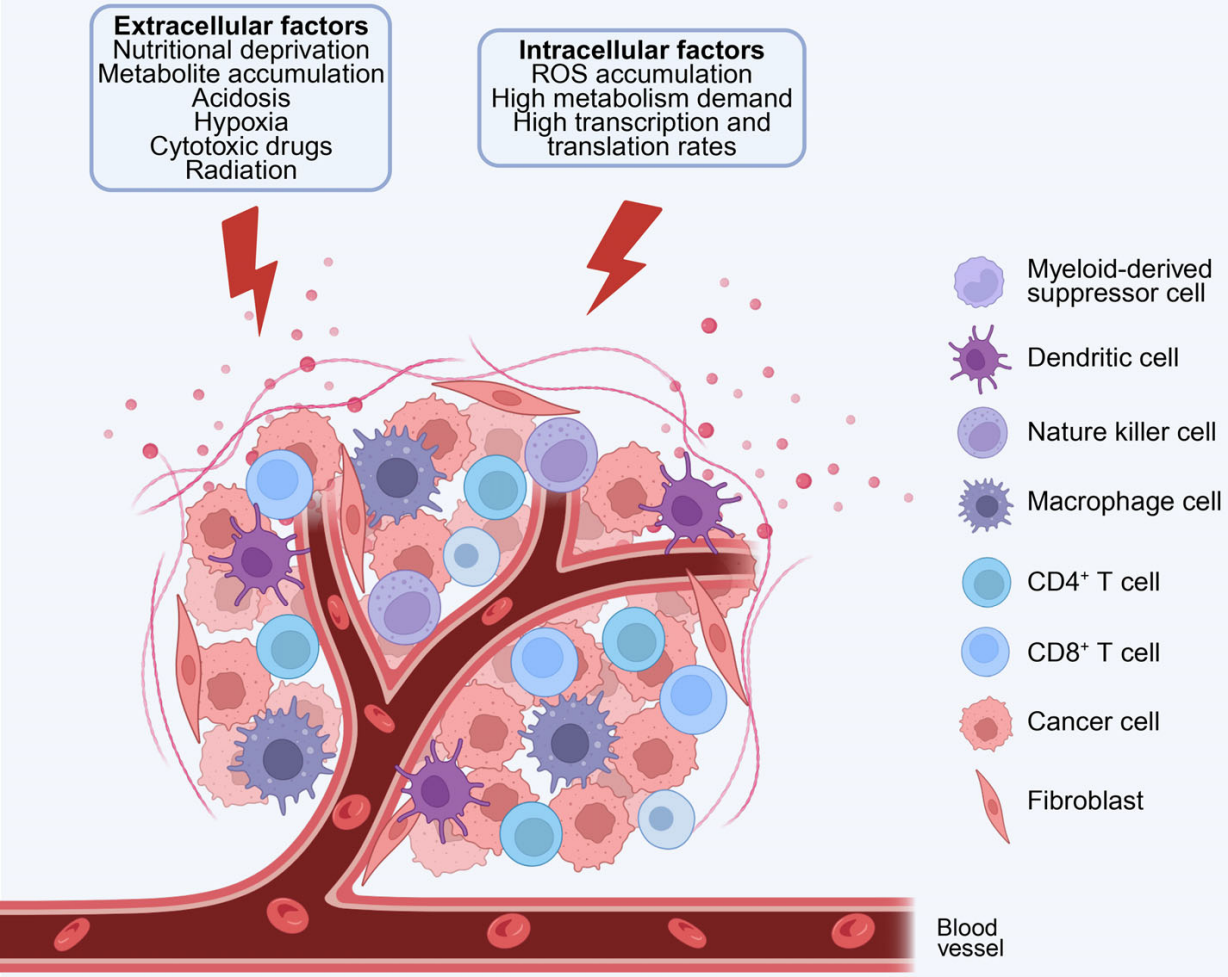
Cells inside the tumor microenvironment encounter several difficulties. The tumor microenvironment (TME) comprises not only tumor cells but also a variety of immune cells, including T cells, natural killer cells, bone marrow‐derived suppressor cells, dendritic cells, and macrophages. Under physiological conditions, these infiltrating immune cells work together to eliminate tumor cells. However, these cells encounter several detrimental influences from both external and intracellular origins, resulting in protein misfolding and disturbance of endoplasmic reticulum (ER) homeostasis. In the extracellular matrix, detrimental events such as nutritional deprivation, metabolite buildup, acidosis, hypoxia, cytotoxic agents, and radiation induce endoplasmic reticulum stress in the aforementioned cells. Simultaneously, intracellular variables such as reactive oxygen species (ROS) buildup, elevated metabolic demand, increased transcription rate, and heightened translation rate induce ER stress in cells. To accommodate the aforementioned components and alleviate endoplasmic reticulum stress, the unfolded protein response (UPR) was initiated. UPR influences the functionality of tumor‐infiltrating immune cells and modifies the immunogenicity of tumor cells, ultimately resulting in tumor immune evasion.

The unchecked proliferation of malignant cells in cancerous tissues results in deleterious microenvironments marked by elevated metabolic demands, hypoxia, nutritional limitations, acidosis, interstitial hypertension, and the accumulation of reactive oxygen species (ROS) (Figure 1) [22, 23]. Furthermore, the activation of oncogenes and the suppression of tumor suppressor genes in cancer cells exacerbate ER stress by increasing the rates of transcription and translation [24]. The combined impact of these adverse intra‐ and extracellular conditions impairs the protein‐folding capacity of the ER in cancer cells, leading to the accumulation of misfolded or unfolded proteins and the subsequent induction of ER stress [25].

Cells respond to ER stress by activating a series of signaling pathways collectively referred to as the UPR. The primary objective of the UPR is to re‐establish ER homeostasis by decreasing the load of secretory proteins, enhancing the proper folding of ER proteins, inhibiting global protein translation, and facilitating the clearance of aberrant proteins through autophagy and ER‐associated degradation (ERAD) [26]. The UPR exerts a wide range of effects, including the alleviation of protein misfolding and the restoration of calcium homeostasis, lipid balance, and protein trafficking [27]. However, chronic activation of the UPR, due to sustained ER stress in cancer cells and other cells within the TME, plays a crucial role in cancer progression by promoting tumorigenesis and metastasis. The activation of the UPR is mediated by three transmembrane ER‐resident stress sensors: inositol‐requiring enzyme 1 (IRE1α), protein kinase R (PKR)‐like ER kinase (PERK), and activating transcription factor 6 (ATF6), which are respectively encoded by the genes ER to nucleus signaling 1, eukaryotic translation initiation factor 2‐alpha kinase 3 (EIF2AK3), and ATF6 [28, 29]. These sensors share similar activation mechanisms (Figure 1). In a nonstressful state, these molecules form stable complexes with the glucose‐regulated protein 78 (GRP78), thereby maintaining their inactive configuration [19]. However, during ER stress, the accumulation of misfolded or unfolded proteins within the ER lumen surpasses the cell's processing capacity. Consequently, GRP78 exhibits a preferential binding affinity for misfolded proteins, leading to its dissociation from IRE1α, PERK, and ATF6 [30]. This dissociation subsequently activates downstream signaling pathways, which facilitate the restoration of normal protein folding and secretion [31].

### 2.1 Inositol‐Requiring Enzyme 1 α

**FIGURE 2:**
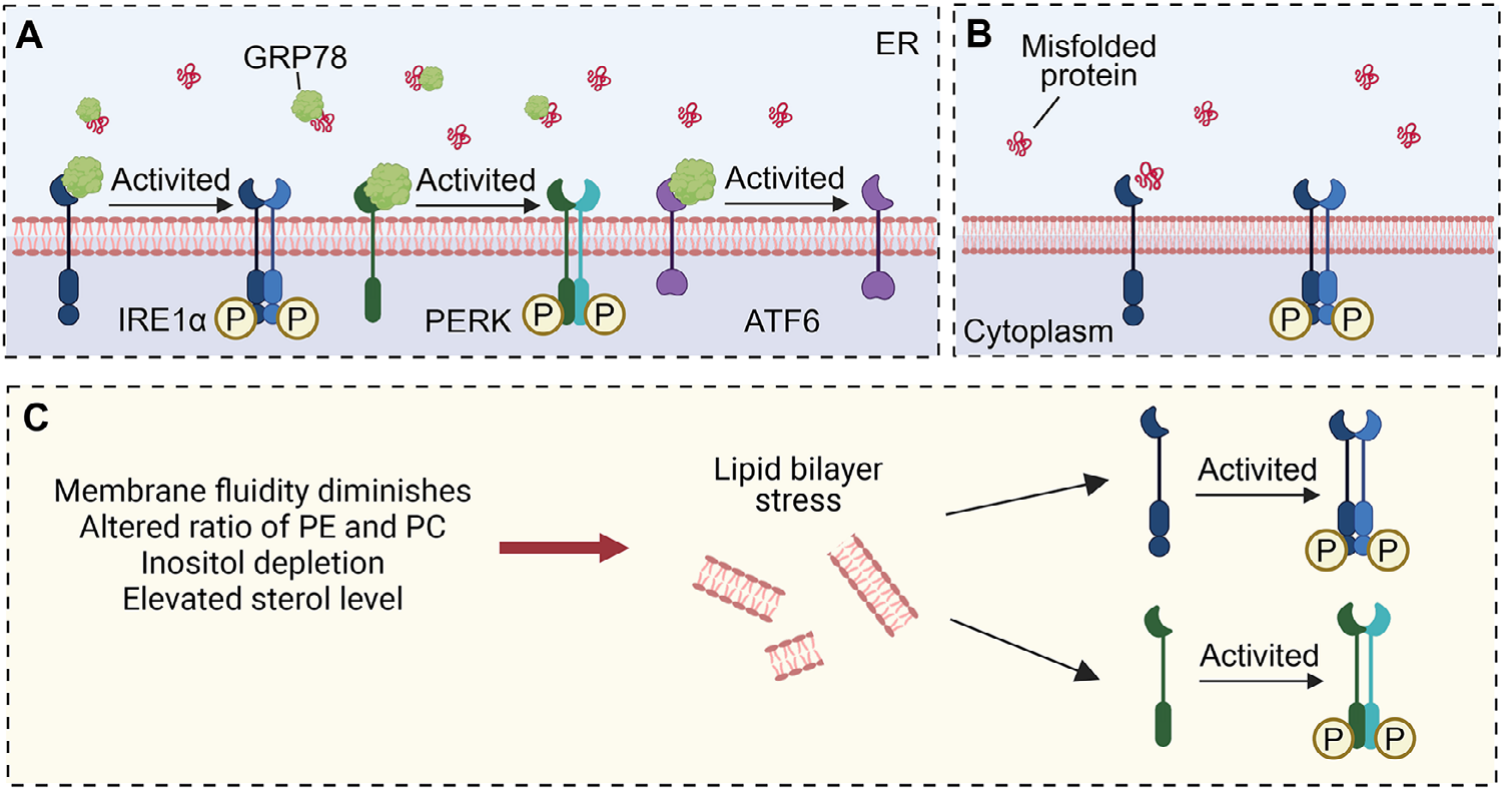
Mechanisms of activation for IRE1α, PERK, and ATF6. (A) In the absence of endoplasmic reticulum (ER) stress, inositol‐requiring enzyme 1 (IRE1α), protein kinase R (PKR)‐like endoplasmic reticulum resident kinase (PERK), and activating transcription factor 6 (ATF6) remain inactive due to their tight association with the glucose‐regulated protein of 78 kDa (GRP78) within the ER. During ER stress, a surplus of misfolded or unfolded proteins accumulates in the ER, leading GRP78 to preferentially associate with these proteins and detach from PERK, ATF6, and IRE1α. This results in the dimerization and autophosphorylation of IRE1α and PERK, therefore activating them. (B) The central ER‐lumenal domain of IRE1α can directly interact with misfolded proteins, initiating its activation. (C) Lipid bilayer stress (LBS) stimulates IRE1α and PERK by activating their respective single transmembrane domains. LBS encompasses reduced membrane fluidity, a modified ratio of phosphatidylethanolamine (PE) to phosphatidylcholine (PC), inositol deficiency, and increased sterol concentrations.

**FIGURE 3:**
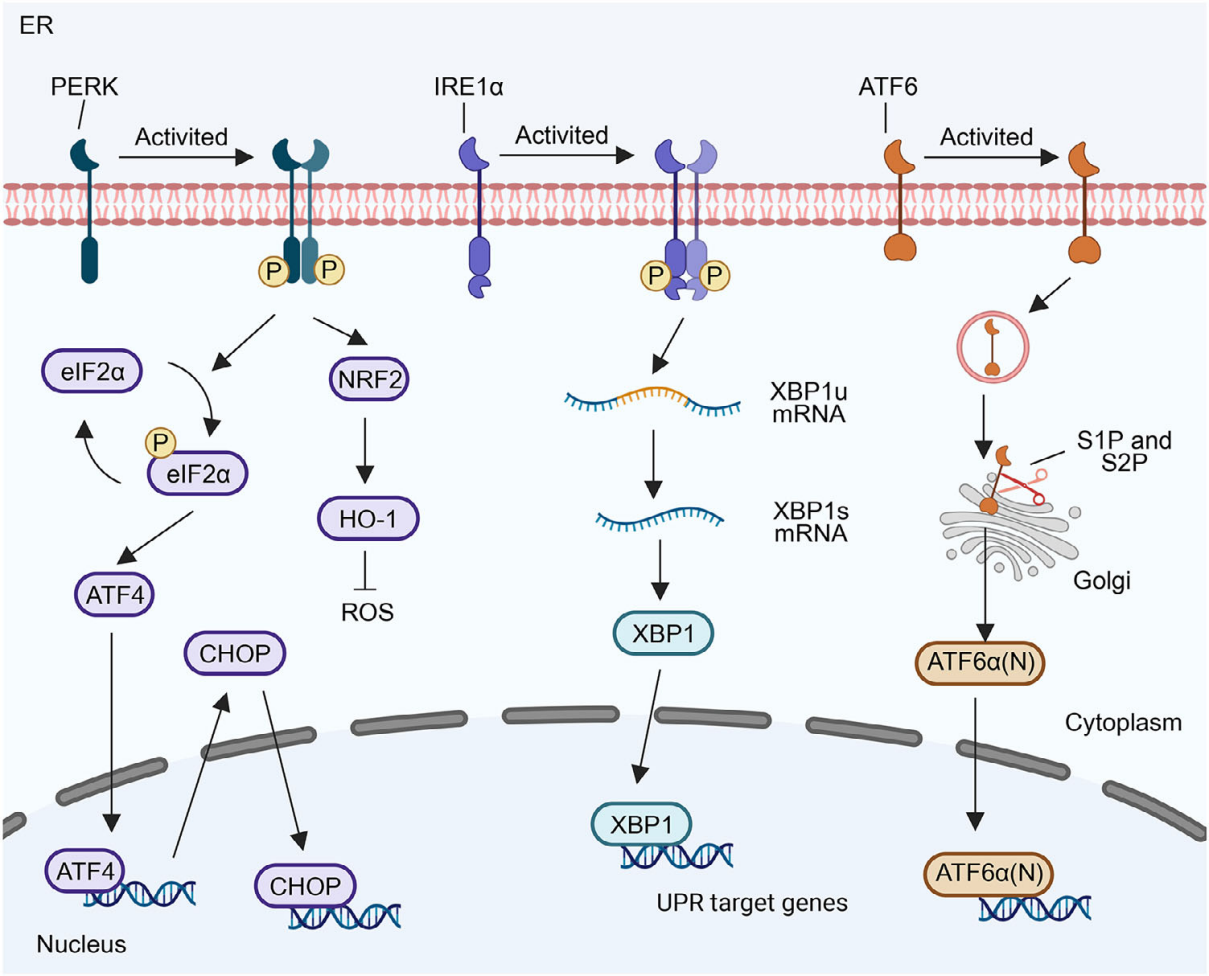
Unfolded protein response occurs when cells experience endoplasmic reticulum stress. The dimerization and autophosphorylation of protein kinase R (PKR)‐like endoplasmic reticulum resident kinase (PERK) activate it and diminish total protein translation via phosphorylating eukaryotic initiation factor 2α (eIF2α). The inhibition of translation upregulates the expression of the transcription factor activating transcription factor 4 (ATF4), which induces the expression of the transcription factor C/EBP homologous protein (CHOP). Inositol‐requiring enzyme 1 (IRE1α) undergoes dimerization and autophosphorylation following its separation from glucose‐regulated protein of 78 kDa (GRP78), leading to the activation of the RNase domain. The RNase domain specifically cleaves the 26‐nucleotide intron of X‐box binding protein 1 (XBP1) mRNA, resulting in the generation of stable and effective XBP1. Upon dissociation from GRP78, the Golgi localization signal of ATF6 is revealed, facilitating its transport to the Golgi apparatus by vesicles. It is cleaved by site‐1 protease (S1P) and site‐2 protease (S2P). The N‐terminal cytoplasmic domain of ATF6α is subsequently translocated from the Golgi apparatus to the nucleus, where it modulates the expression of protein‐folding genes.

In mammals, IRE1 has two subtypes, IRE1α and IRE1β [32]. IRE1α, a type I transmembrane protein, is expressed on ER membranes in all cells. It possesses serine/threonine kinase and ribonuclease (RNase) structural domains at the end of the cytoplasmic domain, exhibiting dual enzyme activities. IRE1α self‐regulates upon dissociation from GRP78, leading to subsequent trans‐autophosphorylation and dimerization mediated by disordered regions in the IRE1α ER lumenal domain [33]. In addition, lipid bilayer stress can also activate IRE1α by stimulating its single transmembrane domain, which includes decreased membrane fluidity, altered ratio of phosphatidylethanolamine and phosphatidylcholine, inositol depletion and elevated sterol level [34]. IREIα also senses ER stress by directly binding to misfolded proteins (Figure 2) [35]. These events induce a conformational change, activating the RNase structural domain of IRE1α. The RNase catalyzes the removal of the 26‐nt intron in unspliced X‐box binding protein 1 (XBP1) mRNA (XBP1u), producing spliced XBP1 (XBP1s), which is a transcription factor [36]. XBP1s upregulate various protein folding‐related enzymes, oxidoreductases, intracellular transport components, ER‐related degradation mechanisms, lipid synthesis, and glycosylases to restore ER homeostasis (Figure 3) [37].

IRE1β shares similar structures and functions with IRE1α. IRE1β has different RNase structural domains with IRE1α, leading to weaker XBP1‐splicing potential [38]. Unlike widely distributed IRE1α, IRE1β is only expressed in epithelial cells of the respiratory and gastrointestinal tracts, where it maintains mucosal homeostasis [39]. This distinct expression pattern between IRE1α and IRE1β might be related to microbial colonization [40]. IRE1β can be induced by intestinal microorganisms and, upon activation, alleviates ER stress and UPR in goblet cells. It promotes goblet cell maturation and regulates colon mucus barriers through XBP1 splicing and the regulated IRE1‐dependent decay (RIDD) pathway. These mucus barriers provide a niche for colonizing microbes, which product butyrate to upregulation IRE1β level [40]. Interestingly, IRE1β can bind IRE1α, inhibiting XBP1 splicing in response to ER stress. This weakened UPR maintains goblet cell homeostasis and mucosal defense.

### 2.2 Protein Kinase R‐Like ER Kinase

PERK, a type I transmembrane kinase, has the same activation mechanism as IREIα (Figure 2). Phosphorylation of activated PERK at serine 51 inhibits 5′cap‐dependent mRNA translation via eukaryotic initiation factor 2α (eIF2α), reducing new protein influx into the ER [41]. This restriction aids the centralized refolding of ER‐localized chaperones by limiting overloaded protein entry into stressed cell ERs. Paradoxically, translation inhibition increases selective translation of the transcription factor ATF4, thereby increasing the expression of the transcription factor C/EBP homologous protein (CHOP) [42]. ATF4 and CHOP synergistically induce the expression of multiple genes involved in amino acid biosynthesis, protein folding, autophagy, redox homeostasis, amino acid metabolism, and cell apoptosis (Figure 3). The PERK signaling cascade activates adaptive pathways in cancer rather than inducing cancer cell death. Notably, PERK is upregulated in various cancers, including the brain, central nervous system [43], breast, pancreatic ductal adenocarcinoma [44], and hepatocellular carcinoma (HCC) [45]. This upregulation correlates with unfavorable prognostic outcomes in these cancers [46].

### 2.3 Activating Transcription Factor 6 α

In contrast to IRE1α and PERK, ATF6 is a type II ER transmembrane protein with a cytoplasmic domain containing a bZIP transcription factor. ATF6 has two isoforms in mammals: ATF6α and ATF6β, of which ATF6α is mainly involved in transcriptional regulation [47]. Unlike IRE1α and PERK pathways, ATF6α, during ER stress, is transported from the ER to the Golgi apparatus via vesicles containing coat protein II (COPII) [48]. Within the Golgi apparatus cavities, ATF6α is first cleaved into two halves by site‐1 protease (S1P). One half, with its NH2 end still anchored to the membrane, is further cleaved by site‐2 protease (S2P). Subsequently, the N‐terminal cytoplasmic domain of ATF6α dissociates from the cell membrane and translocates into the nucleus, activating ER stress‐related genes [49]. Cleaved ATF6α regulates the expression of genes involved in protein folding, ERAD, and activates XBP1 genes in an ER stress‐response element‐dependent manner [50]. The cytoplasmic domain of ATF6 can form a heterodimer with XBP1, inducing the expression of genes related to protein folding and ER degradation, such as molecular chaperones, foldases, and components of the ERAD system (Figure 3).

### 2.4 Glucose‐Regulated Protein **78**

GRP78, also known as binding immunoglobulin protein, is a member of the heat shock protein 70 superfamily [51]. It comprises a nucleotide‐binding domain (NBD) and a substrate‐binding domain (SBD). As a critical ER‐resident chaperone, GRP78 participates in UPR regulation through complex mechanisms. Despite extensive research on the interaction between GRP78 and IRE1α, two contradictory models have emerged. In the first model, the luminal stress‐sensing domain of IRE1α (IRE1α LD) binds to the GRP78 SBD. ER‐localized J‐protein 4 (ERdj4) selectively binds IRE1α through its C‐terminal domain and promotes its interaction with GRP78 SBD by enhancing the ATPase activity of the GRP78 NBD. The GRP78–IRE1α complex inhibits IRE1α dimerization, suppressing UPR activation. Nucleotide exchange factors promote adenosine diphosphate (ADP) and ATP turnover in the NBD, triggering IRE1α release from the SBD. However, during ER stress, the increased accumulation of misfolded proteins competes with IRE1α for GRP78 SBD binding, leading to IRE1α dimer formation and the UPR pathway activation [52]. Notably, ATP induces GRP78 dissociation from IRE1α but does not affect misfolded protein–GRP78 interaction [53]. In the second scenario, IRE1α’s LD binds to the NBD of GRP78, remaining unaffected by nucleotides. During ER stress, misfolded proteins bind to the SBD of GRP78, inducing a conformational change in the NBD, leading to the dissociation of IRE1α’s LD from GRP78. Due to the strikingly similar structures of IRE1α LD and PERK LD, GRP78 regulates PERK like IRE1α [54]. In contrast, ATF6 exhibits a distinct structural configuration from IRE1α and PERK, suggesting a potentially different regulatory mechanism for GRP78 on ATF6. However, the precise mechanism underlying the formation and dissociation of the GRP78–ATF6 complex requires further elucidation. Studies have demonstrated the stability of the GRP78–ATF6 complex, indicating that misfolded proteins are ineffective in competing with GRP78 to dissociate the complex within stressed cells [55].

Moreover, the Lys–Asp–Glu–Leu sequence at the C terminal of GRP78 is associated with its ER localization and function [56]. However, in the TME, GRP78 is evaluated in long‐term stressed cancer stem cells, cancer‐associated endothelial cells, and cancer cells, leading to its translocation to the cell surface. These cell surface GRP78 (csGRP78) molecules promote cancer cell proliferation, metastasis, and angiogenesis while inhibiting apoptosis [57]. Furthermore, csGRP78 contributes to cancer cell resistance to drugs, reducing cancer cell sensitivity during treatment [58]. Remarkably, csGRP78 is absent in normal cells, making it a promising target for cancer therapy [59]. Targeting csGRP78 offers the potential to selectively eliminate cancer cells without substantial toxic effects on normal cells.

csGRP78 is ultimately secreted into the environment and is referred to as secreted or soluble GRP78 (sGRP78). sGRP78 belongs to the resolution‐associated molecular patterns and regulates immune cell function to achieve inflammation resolution [60]. sGRP78 promotes apoptosis and ferroptosis of myeloid cells [61]. sGRP78 facilitates the conversion of splenic B cells into regulatory IL‐10^+^PD‐L1^hi^FasL^hi^ B cells [62] and bone marrow‐derived dendritic cells (BMDCs) into tolerogenic dendritic cells (DCs) characterized by diminished MHC‐II expression and elevated levels of B7‐H3 and B7‐H4 [63]. Additionally, in LPS‐conditioned BMDCs and BMDMs, sGRP78 significantly suppresses the secretion of inflammatory cytokines, such as IFNβ, IL1β, IL6, and TNFα [64]. sGRP78 stimulate M2 polarization in macrophages, evidenced by the overexpression of CD206 and the downregulation of CD80 [65]. In summary, sGRP78 facilitates a transition in the immune microenvironment toward tolerance. This shift not only relates to inflammatory illnesses but also promotes the proliferation and metastasis of cancer cells following immune evasion [66].

## 3 The Role of ER Stress in Cancer Progression

In cancer, the UPR is often activated to support tumor growth and survival. Tumor cells, which are characterized by rapid proliferation and high metabolic demands, frequently experience ER stress due to the increased need for protein synthesis and the harsh conditions of the TME, such as hypoxia and nutrient deprivation. The UPR helps cancer cells adapt to these conditions by enhancing their protein‐folding capacity and promoting survival pathways. For instance, the activation of UPR signaling pathways, including IRE1, PERK, and ATF6, has been shown to support tumor growth and metastasis by promoting cell survival, angiogenesis, and immune evasion.

### 3.1 UPR Promotes Tumor Proliferation and Metastasis

Tumor proliferation, invasion and migration are regulated by complex networks. UPR has been found to crosstalk with multiple tumor‐related signaling pathways to promote tumor progression.

In triple‐negative breast cancer (TNBC), the inhibition of XBP1 significantly impedes TNBC growth and metastasis to the lungs. This effect is not attributed to alterations in apoptosis, cell proliferation (Ki67), or the overactivation of IRE1 and other branches of the UPR. Instead, it results from XBP1's regulation of the transcriptional program of HIF1α, which mediates the hypoxia response pathway [14]. There exists a bidirectional crosstalk between UPR and mTOR signaling [67]. The upregulation of protein synthesis mediated by mTOR leads to the accumulation of misfolded or unfolded proteins in the ER lumen, thereby inducing ER stress. Conversely, UPR can enhance autophagy by attenuating the mTOR pathway [68]. Furthermore, the interaction between MAPK and UPR can also result in the accumulation of autophagosomes in lung cancer, ultimately leading to cell death [69]. However, PERK appears to play an opposing role. In both AML and colorectal cancer, PERK activation has been observed to induce autophagy in tumor cells [70, 71], potentially through the accumulation of ROS. In addition to autophagy, UPR is also closely related to tumor cell apoptosis. It is generally believed that the continuous activation of UPR signals will eventually lead to cell death. PERK can activate NRF2, which can bind to ARE and upregulate the expression of antiapoptotic proteins BCL‐2 and Bcl‐xL [72]. At the same time, excessive UPR can also induce apoptosis in a p53‐dependent manner [73]. In gastric cancer, UPR is activated after GRP78 is polyubiquitinated and degraded, leading to caspase‐dependent apoptosis [74]. Ca^2+^ plays an important role in the process of UPR‐induced apoptosis. ER stress leads to dysregulation of ER Ca^2+^ homeostasis, resulting in excessive accumulation of Ca^2+^ in mitochondria, increasing mitochondrial membrane permeability and promoting the release of cytochrome C, ultimately leading to apoptosis [75].

Tumor metastasis is intricately associated with epithelial‐to‐mesenchymal transition (EMT), and there is a reciprocal influence between EMT and the UPR. Within cells undergoing EMT, the PERK–eIF2α signaling axis is preferentially activated over other pathways [76]. Importantly, the activation of PERK is essential for the invasive and metastatic capabilities of EMT cells. PERK effectively prevents apoptosis during the loss of intercellular contact caused by EMT, while promoting migration and tumor sphere formation [76, 77]. Furthermore, PERK contributes to the promotion of EMT by enhancing the nuclear translocation of ATF4 and augmenting the transcriptional activity of the interleukin (IL)‐like EMT inducer [78].

### 3.2 UPR Contributes to Tumor Angiogenesis

Solid tumors require the formation of new blood vessels, a process known as angiogenesis, to support their growth and survival by ensuring an adequate supply of nutrients and oxygen, as well as the removal of metabolic waste. This process is crucial for tumor progression and metastasis, as it allows tumors to expand beyond their initial size and invade surrounding tissues. Angiogenesis is driven by a complex interplay of proangiogenic and antiangiogenic factors, with vascular endothelial growth factor (VEGF) being one of the most significant proangiogenic signals [79]. Recent research has demonstrated that the UPR significantly facilitates tumor angiogenesis. Specifically, transcription factors such as ATF4, XBP1, and ATF6 have been shown to interact with the VEGF promoter, thereby enhancing VEGF expression and promoting endothelial cell proliferation, survival, and angiogenesis [80]. In the context of medulloblastoma, activation of the UPR triggers the production of VEGF, FGF2, angiopoietin, and IL8, while also stabilizing VEGF mRNA [81]. Inhibition of the IRE1 pathway results in the downregulation of several proangiogenic factors and enhances the efficacy of anti‐VEGF therapy, which is generally ineffective as a standalone treatment in TNBC [82, 83]. Similarly, PERK inhibition markedly suppresses tumor growth and angiogenesis in an orthotopic squamous cell carcinoma model by reducing the expression of FGF2, VEGF, and IL‐6, while increasing the levels of antiangiogenic cytokines/chemokines such as thrombospondin 1 (THBS1), CXCL14, and CXCL10 [84].

### 3.3 The Role of UPR in Immunoediting of TME

Cancer cells, arising from mutations in normal cells, express various tumor antigens (TAs) that activate the immune system [85]. This immune response can eliminate cancer cells or shape cancer immunogenicity, inhibiting anticancer immunity and aiding cancer progression [86]. This dual role of the immune system in cancer growth is referred to as cancer immunoediting, describing the dynamic processes controlling cancer development. Cancer immunoediting encompasses three distinct stages: elimination, equilibrium, and escape [87]. The immune system identifies, surveils, and eliminates most cancer cells. However, certain cancer cells evade clearance, entering an “equilibrium” state where intricate interactions occur between the immune system and cancer cells, often without clinical symptoms. In severe cases, this equilibrium can persist for a lifetime. Once cancer cells breach this equilibrium state, they successfully achieve immune escape, causing the immune system to lose control over them [88].

The entire cancer immunoediting process is closely related to various infiltrating innate and adaptive immune cells in the TME. Innate immune cells, such as natural killer (NK) cells, eosinophilic granulocytes, basophilic granulocytes, and phagocytes (mast cells, neutrophilic granulocytes, monocytes, macrophages, and DCs), inhibit cancer by directly killing cancer cells or inducing adaptive immune responses [89]. Adaptive immunity involves antigen‐presenting cells (APC) like DCs, which capture antigens on cancer cell surfaces and present them to CD8^+^ T cells and CD4^+^ T cells through major histocompatibility complex (MHC)‐I class and MHC‐II class pathways [90]. Successful anticancer immunity relies on the efficient capture of TAs by APCs and subsequent presentation to T cells, particularly CD8^+^ T cells, activating them to recognize and eradicate cancer cells [91]. During cancer immunoediting, immune cells have a dual function, acting as guardians (anticancer immunity) or supporters of cancers (procancer immunity).

In response to ER stress, cancer cells and TIICs adaptively trigger the UPR, allowing them to overcome diverse challenges within the TME. Activation of the UPR signaling pathway can promote cancer cell immune escape by regulating various stages of these immune processes, including expressions, TA presentation, antigen uptake, cross‐presentation by APCs, and immune cell function.

#### 3.3.1 UPR Downregulates the Antigen–MHC‐I Complex in Cancer Cells

**FIGURE 4:**
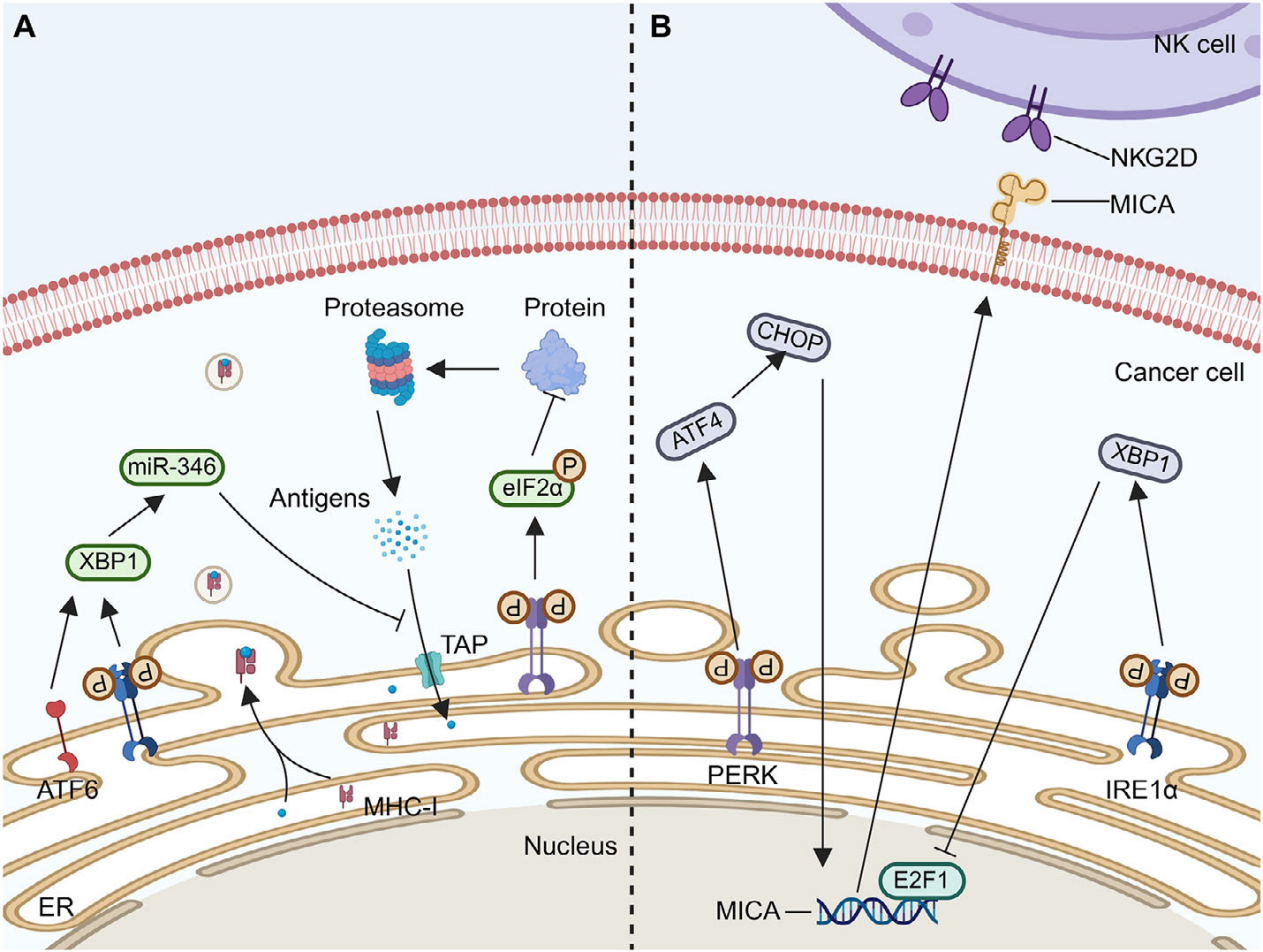
Effects of endoplasmic reticulum stress on antigen–MHC‐I complex and natural killer cells. (A) Tumor cells generate several tumor antigens by the proteolytic degradation of proteins. Tumor antigens translocate into the endoplasmic reticulum (ER) through antigen processing‐associated transporters (TAP) and connect with major histocompatibility complex (MHC) class I molecules. During ER stress, protein kinase R‐like endoplasmic reticulum kinase (PERK) triggers the downstream eIF2α–ATF4–CHOP signaling cascade and diminishes the expression of MHC‐I on the cell surface. The phosphorylation of eIF2α results in a reduction in protein translation, thereby impacting the quantity of antigenic peptides. As a result, there is a decreased amount of antigenic peptides binding to MHC‐I in the endoplasmic reticulum, affecting their location within the ER. Both inositol‐requiring enzyme 1 alpha (IRE1α) and activating transcription factor 6 (ATF6) can enhance the expression of X‐box binding protein 1 (XBP1), consequently elevating miR‐346 expression levels. The direct targeting of TAP by miR‐346 leads to a reduction in its expression, thereby diminishing the transfer of antigenic peptides to the endoplasmic reticulum and their binding to MHC‐I. (B) The IRE1α–XBP1 pathway dampens the expression of MICA and E2F1, which serves as a regulator of MICA promoters. Moreover, the loss of E2F1 binding site in MICA resulted in the loss of sensitivity to ER stress. In contrast, the PERK–ATF4–CHOP signaling pathway elevates MICA levels.

TAs comprise tumor‐specific antigens (TSAs) and tumor‐associated antigens (TAAs) [92]. TSAs are unique to cancer cells and are ideal targets for T cell‐based immunotherapy due to their absence in normal cells. Consequently, they offer ideal immune targets with enhanced therapeutic specificity and reduced risk of nonspecific autoimmunity [93, 94]. TAs can bind MHC molecules at different locations in cells. In the cytoplasm, TAs are regulated by 26S proteasomes, generating short peptides transported to the ER by a transporter associated with antigen processing (TAP) for binding with MHC‐I. Exogenous TAs enter this pathway through phagosome reverse transport. Despite abundant antigen expressions, most cancers evade immune system‐mediated damage. ER plays a crucial role in antigen–MHC‐I complex generation. Proper ER function affects TA recognition by immune cells. UPR activation under ER stress affects complex production, facilitating cancer cell immune escape (Figure 4A). In various cancers such as thyroid cancer [95], non‐small cell lung cancer [96], and colorectal cancer [97], downregulation of MHC‐I enhances cancer cell immune escape, correlating with poor prognostic outcomes [98].

Activation of the UPR in cancer cells reduces MHC‐I on cell membranes, primarily through the PERK pathway [99]. ER stress from palmitate or glucose deprivation triggers the UPR, lowering MHC‐I expression on lymphoma cells without affecting intracellular levels. PERK phosphorylates eIF2α, inhibiting translation and reducing antigen peptide supply, causing some MHC‐I molecules to remain in the ER. [100]. Disseminated cancer cells (DCCs) in the liver of pancreatic cancer patients and mice models show ER stress with PERK overactivation and increased CHOP expression, but not IRE1α involvement. Reducing ER stress with 4‐PDA in mouse liver decreases DCCs and increases MHC‐I on cell surfaces [101]. The activation of the PERK–CHOP pathway in the UPR reduces MHC‐I expression on pancreatic cancer cells, hindering CD8+ T cell cytotoxicity and aiding metastasis.

It has also been suggested that XBP1s alter the TA–MHC‐I complex rather than the PERK signaling pathway. In hepatitis B virus (HBV)‐infected hepatoma cells, ethanol‐induced ER stress activates ATF6 and IRE1α, disrupting HBV core peptide–MHC‐I transport and impairing CD8+ T cell recognition [102]. Overexpressed XBP1 targets TAP1, reducing its expression via miR‐346, preventing TA–MHC‐I complex assembly [103]. Overall, ATF6, IRE1α, and PERK pathways may impair TA–MHC‐I complex function.

The PERK axis primarily decreases surface complex formation, whereas ATF6 and IRE1α pathways mainly regulate the transport of the complexes. The specific activation of the UPR signaling pathway appears to be influenced by multiple factors, including cancer type and cell line source, which impact UPR protein expression. The search for substances capable of selectively stimulating the activation of a particular UPR pathway is an area that requires further exploration.

#### 3.3.2 UPR Modulates NK Cell Activity and Cytotoxicity

NK cells, part of the innate immune system, effectively manage antiviral and anticancer responses without needing prior antigen activation. Unlike other immune cells, they do not rely on MHC restriction or specific antigen recognition. Their activity is controlled by a balance of activating and inhibitory signals from receptor–ligand interactions. Key activating receptors include AKIR, NCR, and NKG2C/D, while inhibitory receptors like IKIR and NKG2A modulate their responses. [104]. The interaction between NKG2D and its ligand (NKG2DL) determines NK cells’ ability to kill target cells. NKG2DL includes the MHC‐I chain‐related (MIC) gene family (MICA and MICB) and the UL16 binding protein (ULBP) family (ULBP1‐6). Enhancing NK cells’ frequency, infiltration, and functions is associated with better patient survival rates. [105]. NK cells kill cancer cells by releasing perforin and granzyme and triggering apoptosis through Fas/FasL and tumor necrosis factor (TNF)‐related apoptosis‐inducing ligand pathways [106]. They also produce proinflammatory cytokines and chemokines like IFN‐γ, TNF, IL‐6, granulocyte macrophage‐colony stimulating factor (GM‐CSF), and C‐C Motif Chemokine Ligand 5 (CCL5) to enhance anticancer effects [107]. Although effective against circulating cancer cells, their function is impaired in the TME due to the immaturity of cancer‐infiltrating NK cells, resulting in decreased cytotoxicity [13].

The hostile TME can regulate the anticancer effects of NK cells by modifying the activity and expression levels of receptors on NK cell surfaces (Figure 4B). In conditions such as multiple myeloma, the hypoxic microenvironment dampens NK cell degranulation and downregulates the expressions of NKG2D, perforin, and granzyme B, thereby weakening NK cell‐mediated cytotoxicity [108]. In melanoma, the IRE1α–XBP1 axis inhibits the E2F1 binding site on the MICA promoter, consequently suppressing MICA expression, while the expression of ULBP proteins remains unaffected. This lack of E2F1 binding site renders melanoma cells insensitive to ER stress [109].

Apart from the IRE1α axis, the PERK–ATF4–CHOP pathway upregulates explicitly the expression of NKG2DL (mainly MICA and ULBP2) in cancer cells [110], enhancing their binding to NKG2D and subsequently activating NK cells for cancer cell destruction. However, studies have shown that ATF4 can directly bind to the ULBP1 promoter [110], leading to its transactivation, with minimal effects on other NKG2D ligands. Furthermore, cancer cells expressing high levels of NKG2DL can release soluble NKG2DL via exosomes, downregulating NKG2D expressions and preventing NK cell activation, thereby reducing NK cell cytotoxicity [111].

Although the UPR has been implicated in NKG2D regulation, the mechanisms enabling selective NKG2D regulation warrant further exploration. Moreover, the potential role of UPR in regulating the immaturity of NK cells within the TME remains unknown.

#### 3.3.3 UPR Inhibits TA Presentation by Dendritic Cells

As mononuclear phagocytes, DCs act as professional APCs in the TME [112]. DCs originate from bone marrow pluripotent hematopoietic stem cells, manifesting in various phenotypes in the human body, including conventional DCs (cDC) with subsets of cDC1 and cDC2 [113], plasma cell‐like DCs, myeloid DCs (mDC), inflammatory DCs, and Langerhans cells [114]. Mature immunogenic DCs play a crucial role in antigen presentation and provide costimulatory signals for T cell activation, which are essential for initiating and progressing anticancer immune reactions [115].

**FIGURE 5:**
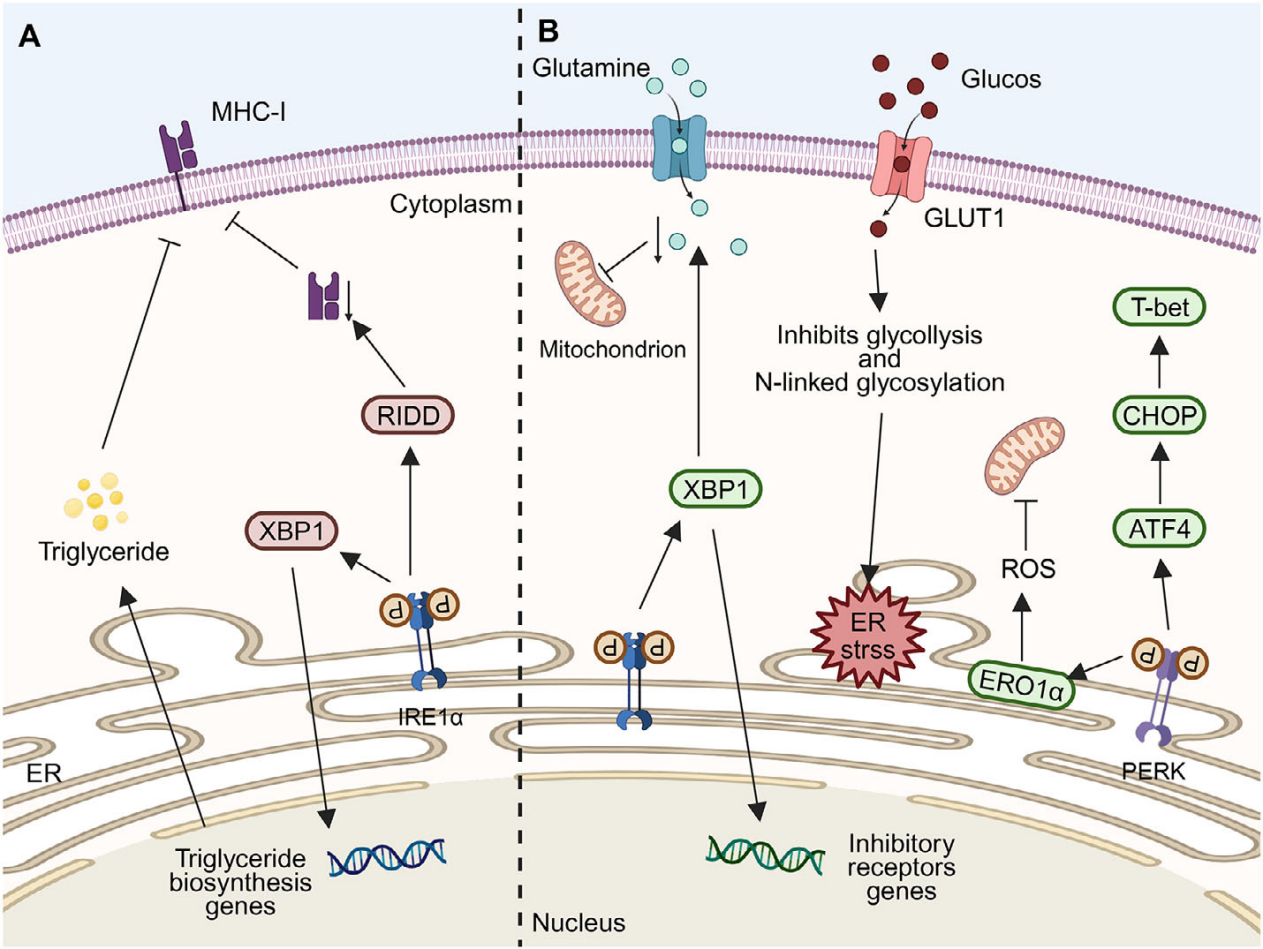
Endoplasmic reticulum stress affects the function of dendritic cells and T cells, leading to immune evasion. (A) In DC cells, the activation of the inositol‐requiring enzyme 1 α (IRE1α)–X‐box binding protein 1 (XBP1) pathway enhances the transcription of many triglyceride production genes. This results in a substantial rise in lipid droplets and total triglyceride levels in the cytoplasm, thereby diminishing the capacity of dendritic cells to process and present antigens. Furthermore, antigenic peptides can directly activate IRE1α and diminish major histocompatibility complex I (MHC‐I) heavy chain mRNA via regulated IRE1‐dependent decay (RIDD), leading to decreased antigen presentation. (B) Glucose transporter type 1 (GLUT1) diminishes on both CD8+ T cells and CD4+ T cells within the tumor microenvironment (TME). This leads to decreased glucose absorption by T cells, inhibiting glycolysis, affecting N‐junction protein glycosylation, and causing endoplasmic reticulum stress. The activation of the IRE1α–XBP1 pathway obstructs glutamine inflow, resulting in the inhibition of mitochondrial respiration. XBP1 simultaneously upregulates the expression of many inhibitory receptors, resulting in the attenuation of the antitumor activity of CD8+ T lymphocytes. The protein kinase R‐like endoplasmic reticulum kinase (PERK)–activating transcription factor 4 (ATF4)–C/EBP‐homologous protein (CHOP) pathway was activated, resulting in the direct inhibition of T‐bet levels. PERK also directly stimulates endoplasmic oxidoreductase 1 α (ERO1α), leading to a substantial elevation in reactive oxygen species (ROS) levels. The buildup of reactive oxygen species (ROS) leads to mitochondrial dysfunction, impairing the functionality of CD8+ T lymphocytes.

Effective anticancer immunity is linked to the number and function of cancer‐infiltrating DCs. ER stress disrupts DCs, impairing antigen presentation and weakening immunity, which allows cancer to grow and spread (Figure 5A). Studies show higher UPR‐related protein levels in TME‐associated DCs than in spleen DCs, with the IRE1α–XBP1 axis playing a key role [116]. In ovarian cancer models, DCs lacking XBP1 slowed cancer progression. In these models, ROS and lipid peroxidation by‐products, such as 4‐HNE, along with GRP78, trigger ER stress in DCs, leading to IRE1α overactivation and increased lipid droplets and triglycerides in DCs. This can be attributed to the regulation of various triglyceride biosynthesis‐related genes like 1‐acylglycerol‐3‐phosphate O‐acyltransferase (Agpat6), fatty acid synthase (Fasn), stearoyl‐CoA desaturase 2 (Scd2), and lysophosphatidic acid receptor 1 (Lpar1) by XBP1 [117]. This leads to lipid accumulation, which hinders antigen processing and T‐cell activation [118]. In pancreatic cancer, valproic acid causes ER stress in DCs by increasing PGE2 release from cancer cells, activating this process [119]. Without ER stress, antigen peptides can activate IRE1α in DCs, depleting MHC‐I mRNA and suppressing antigen cross‐presentation [120].

Transmissible ER stress (TERS) prompts similar responses in DCs as in macrophages, causing DCs to morph into mDCs with larger cell size and thinner processes. It increases the expression of MHC‐I, MHC‐II, and costimulatory molecules (CD68 and CD80) [121]. Although TERS activates and matures DCs, it hinders their ability to cross‐present antigens and activate CD8+ T cells. This might be due to Arg1 secretion and inadequate antigen presentation, leading to reduced CD8+ T cell proliferation, weakened T cell anticancer immunity, enhanced cancer growth, and temporary cancer cell proliferation [121].

#### 3.3.4 UPR Influences the Anticancer Functions of T Cells

T cells, including CD4^+^ and CD8^+^ subsets, play crucial roles in regulating adaptive immunity against cancers and identifying and eliminating cancer cells [122]. CD8^+^ cytotoxic T lymphocytes (CTL) are the preferred immune cells that target cancer [123]. These cells recognize intracellular antigens presented by MHC‐I molecules on various cancer cells. CD8^+^ T cells mediate cancer cell killing through mechanisms involving granzyme and perforin expression and secretion of cytokines such as interferon γ (IFN γ) and TNFα [124]. Additionally, CTLs activate the hypoxia‐inducible factor‐1α (HIF‐1α)/VEGF‐A axis, inhibiting angiogenesis [125]. CD4^+^ T cells differentiate into diverse T helper (Th) cell subtypes, including Th1, Th2, Th9, Th17, and T regulatory cells (Treg), ensuring strict immune regulation. Both CD4^+^ T cells and CD8^+^ T cells identify antigens on the same APCs [126]. CD4^+^ T cells promote APC maturation by interacting with CD40/CD40L, activating CTLs [127], and regulating CD8^+^ T cell proliferation via CD27/CD70 [128]. Precise antigens and CD4^+^ T cell interactions optimize APC antigen presentation, enabling specific cytokine and costimulatory signal provision to CD8^+^ T cells. This facilitates their proliferation, expansion, and differentiation into effector or memory T cells [124].

TA‐specific T cells quickly lose energy and function within cancers due to various UPR pathways (Figure 5B). Under ER stress, T cells activate PERK, leading to mitochondrial failure in CD8+ T cells via ER oxidoreductase 1 (ERO1α), which raises mitochondrial ROS levels and inhibits their anticancer functions [129]. CHOP, a primary negative regulator of CD8^+^ T cell anticancer functions, is activated through the PERK–ATF4–CHOP axis under ER stress, suppressing the main regulator of T cell functions, T‐bet [130]. Apocynin upregulates the PERK–elF2α–ATF4–CHOP pathway, inducing immunogenic apoptosis of breast cancer cells. This enhances cancer immunogenicity, releasing damage‐related molecular patterns and attracting APCs antigen processing and presentation to CD8^+^ T cells, triggering anticancer immune responses [131].

Besides the PERK axis, the IRE1 axis also plays a role in this process. In ovarian cancer, the induction of ER stress and IRE1α–XBP1 axis activation in both CD8^+^ T and CD4^+^ T cells regulate glutamine carrier levels. This limits glutamine entry into CD8^+^ T and CD4^+^ T cells, necessary for mitochondrion respiration under glucose deprivation, suppressing IFNγ expression and reducing cancer‐infiltrating T cell levels [132]. In cancer tissues with high cholesterol levels, cholesterol upregulates IRE1α expression in CD8^+^ T cells and activates XBP1, increasing inhibitory receptor expression, including PD‐1, lymphocyte‐activation gene 3, T‐cell immunoglobulin domain and mucin domain‐3, 2B4, and CTL antigen 4, leading to the loss of CD8^+^ T cell functions [133].

#### 3.3.5 UPR Influences Cancer Growth by Regulating Macrophage Polarization

**FIGURE 6:**
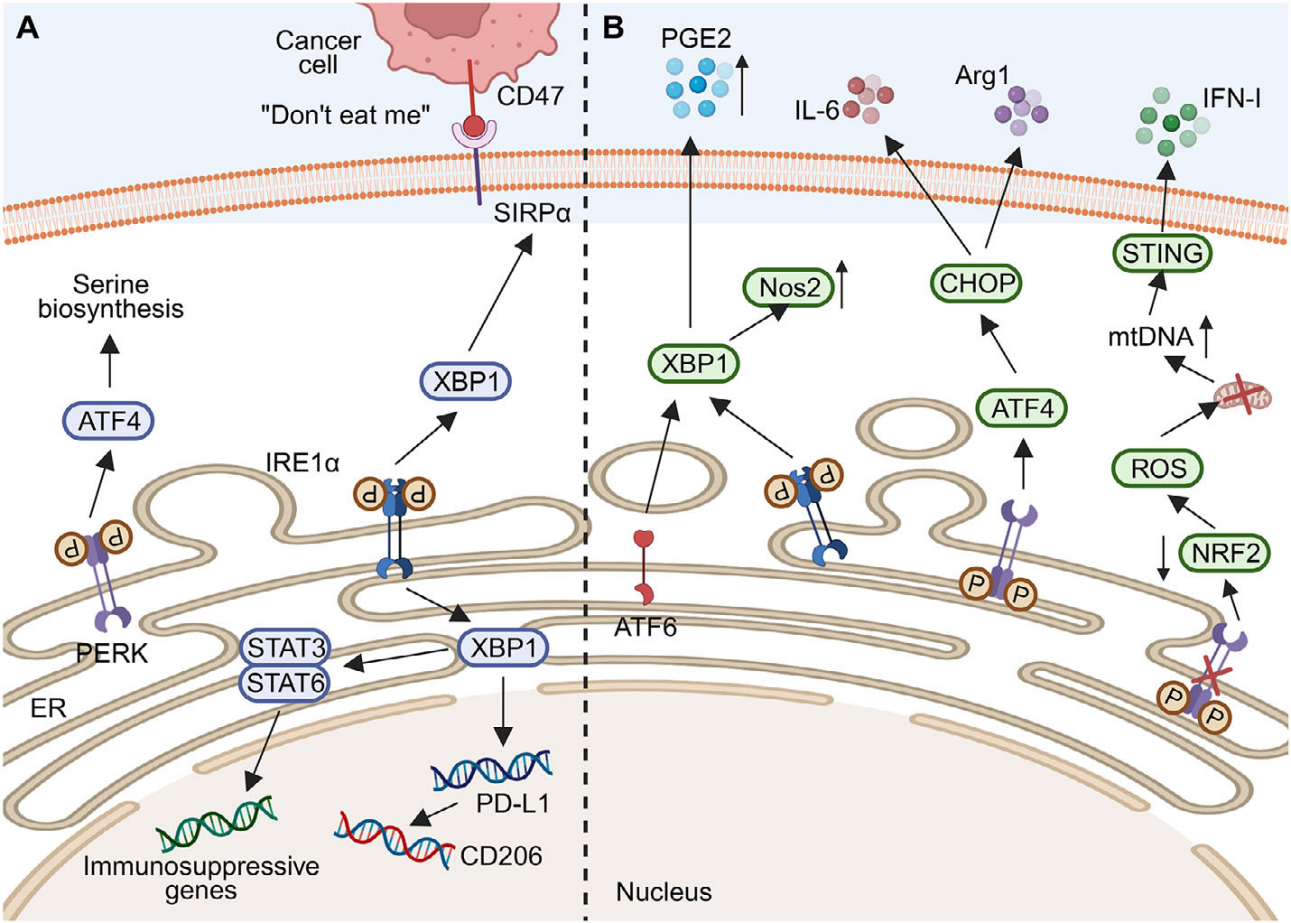
Endoplasmic reticulum stress promotes the immunosuppressive activity of M2 macrophages and myeloid‐derived suppressor cells to enhance tumor immune escape. (A) PERK enhances the expression of ATF4, thereby promoting serine production and facilitating the activation of M2 macrophages. The activation of IRE1α enhances the expression of XBP1 and activates signal transducer and activator of transcription family members 3 and 6 (STAT3/6), promoting polarization toward the M2 phenotype. XBP1 further enhances the expression of signal regulatory protein alpha (SIRPα) on the surface of macrophages. They attach to CD47 on the surface of neoplastic cells to emit the “don't eat me” signal. XBP1 also enhances the expression of PD‐L1. The overexpression of PD‐L1 elevates the amount of CD206, hence predisposing macrophages to the M2 phenotype. (B) IRE1α and ATF6 can both enhance the expression of nitric oxide synthase 2 (Nos2) and facilitate the production of prostaglandin E2 (PGE2) by elevating the amount of XBP1. PERK enhances PMN‐MDSC aggregation by increasing the expression of ATF4 and CHOP. Concurrently, CHOP enhances the production of IL‐6 and arginase 1 (Arg1) to suppress T cell proliferation and function. Besides CHOP, PERK also modulates PMN‐MDSC activity through nuclear factor erythroid 2‐related factor 2 (NRF2). The targeted elimination of PERK leads to a substantial reduction in NRF2 levels, heightened reactive oxygen species (ROS) generation, and mitochondrial dysfunction. Subsequent to mitochondrial depletion, mtDNA levels rise, hence activating the stimulator of interferon genes (STING) pathway. This activation results in the production of IFN‐I and the suppression of NFκB, allowing PERK‐deficient PMN‐MDSCs to perform an antitumor function.

M1 and M2 macrophages constitute tumor‐associated macrophages (TAM). At a high M2/M1 ratio, TAM is associated with poor prognosis of various solid cancers, including gastric cancer [134], thyroid cancer [135], colorectal cancer [136], and breast cancer [137]. The TME promotes macrophage transformation into the M2 phenotype and cancer‐promoting immunity by inducing hypoxia and cytokine secretion, such as IL‐13 [138]. Although hypoxia promotes macrophage polarization toward the M2 phenotype [139], it is not the primary driver of TAM phenotype differentiation, with macrophage polarization being the specific effect of hypoxia [140]. Consequently, the UPR pathway activation in macrophages can promote immune escape by inducing M2 macrophages under ER stress (Figure 6A).

Research has demonstrated that UPR activation in TAMs induces macrophage transformation into the M2 type, exerting immunosuppressive effects. The PERK pathway is pivotal for the immunosuppressive functions of macrophages, promoting M2 polarization in the TME. Decreased PERK levels significantly impair lysosomal functions, oxidative phosphorylation (OXPHOS) in mitochondria, lipid metabolism, glutamine metabolism, and amino acid synthesis in M2 macrophages, thereby affecting their anticancer immunity [141]. Moreover, PERK promotes serine biosynthesis through ATF4, which is essential for M2 macrophage activation [141]. However, it has been demonstrated that PERK is involved in LPS‐induced signal transducer and activator of transcription (STAT) 1 activation, promoting M1 macrophage polarization [142]. The mechanisms underlying this differential effect of PERK on macrophage phenotype remain unclear, possibly influenced by specific factors such as TME conditions and ER stress inducers.

In addition to the PERK pathway, IRE1α also plays a significant role in macrophage transformation into the M2 phenotype. Glucosylceramide, derived from cancer cells, induces unconventional UPR in macrophages by altering lipid composition and saturation on ER membranes, activating IRE1‐mediated splicing XBP1 production and STAT member 3 (STAT3) activation. These events increase M2 macrophage abundance and upregulate the expressions of immunosuppression‐associated genes [143]. Under hypoxia, the inhibition of the IRE1–XBP1 pathway by kinase‐inhibiting RNase attenuator member 6 (KIRA6) promotes glycolysis and inhibits fatty acid oxidation. This reprogramming leads M2 macrophages to repolarize into M1 macrophages, effectively delaying cancer growth and enhancing the efficacy of PD‐1 antibodies [144]. Following infection of macrophages by Kaposi's sarcoma herpesvirus, the polarization of macrophages skews toward the M2 type through the activation of STAT3 and STAT6 pathways. This activation subsequently triggers the IRE1α–XBP1 axis, promoting the expression of PD‐L1 and the secretion of cancer cell‐promoting factors such as IL‐10, VEGF, IL‐6, and IL‐8 [145].

Activation of the UPR can promote cancer progression by regulating cytokine secretion in TAM, with XBP1 playing a crucial role. In mice with colorectal cancer, upregulation of XBP1 activation in macrophages promotes cancer growth and metastasis. XBP1 directly regulates cytokine transcription in macrophages, upregulating IL‐4, IL‐6, and VEGFA expressions. Elevated levels of these cytokines are significantly associated with short‐term prognosis in colorectal cancer patients [146]. Additionally, XBP1 upregulates the transcription of signal regulatory protein α (SIRPα) and THBS1, increasing their presence on macrophage surfaces. This promotes interaction with CD47 on cancer cells, triggering the “don't eat me” signal and preventing cancer cell phagocytosis [146].

GRP78 also plays a vital role in regulating macrophage polarization. GRP78 promotes M2 polarization and inhibits M1 differentiation by activating the JAK/STAT pathway, leading to cell proliferation and migration of lung cancer cells [147]. sGRP78 secreted from liver cancer cells can bind to macrophages, reduce the levels of MHC and CD86, and ultimately lead to M2 polarization [66].

Apart from the UPR activation, cancer cells within the TME can release substances upon their own UPR activation, influencing macrophage function. For instance, when under the UPR, hepatoma cells release exosomes enriched with miR‐23a‐3p. These exosomes alter PD ligand 1 (PD‐L1) expression in macrophages through the phosphatase tension homolog‐serine/threonine protein kinase pathway, increasing CD8^+^ T cell mortality [148]. Similar pathways exist in breast cancer [149] and head and neck cancers [150]. Overexpression of PD‐L1 in THP‐1 macrophages induces CD206 expression, suggesting that the UPR can transform macrophages into the M2 phenotype by upregulating PD‐L1 expression [150]. Cytokines secreted by UPR‐activated cancer cells can affect macrophages in distant locations through blood circulation. Various cancer cells in mice secrete unknown soluble factors under ER stress, upregulating the expression of UPR‐related genes like GRP78, Gadd34, CHOP, and XBP1 in bone marrow‐derived macrophages in a toll‐like receptor 4 (TLR4)‐dependent manner [151]. This ER stress, which is transmitted to bone marrow cells from cancer cells through extracellular effects, is referred to as TERS. The expression of TLR4 promotes the secretion of cancer‐promoting factors by macrophages, including IL‐6, IL‐23p23, and TNF‐α, enhancing inflammation and cancer growth [151]. IL‐23p23 inhibits anticancer immunity mediated by CD8^+^ T cells, CD4^+^ T cells, and NK cells. IL‐6 and IL‐23 contribute to Th17 differentiation, maintain inflammatory environments in the TME, and support cancer growth 151].

#### 3.3.6 UPR Facilitates the Immunosuppressive Effects of Myeloid‐Derived Suppressor Cells

MDSCs, a group of immunosuppressive myelocytes, serve as precursors for DCs, macrophages, and granulocytes. They are divided into two types: polymorphonuclear MDSCs (PMN‐MDSC), which are similar to neutrophilic granulocytes, and mononuclear MDSCs (M‐MDSC), resembling monocytes [152]. Under physiological conditions, they can rapidly differentiate into mature granulocytes, DCs, and macrophages, regulating immune function in organs and tissues. However, during tumorigenesis, cytokines secreted by tumor cells pathologically activate MDSCs, resulting in relatively poor phagocytic activities and continuous production of ROS, nitric oxide (NO) [153], and anti‐inflammatory cytokines [154]. MDSCs, critical participants in cancer immune escape, inhibit immune cell functions and promote cancer metastasis [155], angiogenesis, and EMT [156]. Therefore, MDSCs become essential targets for immunotherapy [157].

The UPR is linked to the immunosuppressive functions of tumor‐associated MDSCs (Figure 6B). While both PMN‐MDSCs and M‐MDSCs show UPR, its activation is crucial only for PMN‐MDSCs’ immunosuppressive roles [158]. Tcyganov et al. [158] reported that contrary to the PERK pathway, the IRE1α and ATF6 pathways directly control the immunosuppressive activities of PMN‐MDSCs. The absence of IRE1α or ATF6 suppresses NO synthase 2 (Nos2) expression in PMN‐MDSCs within tumors, Arg1 expressions in PMN‐MDSCs in the spleen, and PGE2 secretion in the TME, thus promoting antitumor immune responses [158]. These pathways likely affect XBP1 transcription. LOX‐1+PMN‐MDSCs show higher XBP1 and SEC61A expression than LOX‐1+PMN, with LOX‐1 potentially marking human PMN‐MDSCs and supporting their role in hindering antitumor immunity [159].

The PERK axis is crucial in suppressing antitumor immunity via tumor‐infiltrating MDSCs. When MDSCs lack PERK, reduced NRF2 causes ROS buildup and mitochondrial dysfunction, increasing cytosolic mitochondrion DNA (mtDNA). This triggers the stimulator of the interferon genes (STING) pathway, boosting type I IFN expression and aiding antitumor responses [160]. Additionally, the STING pathway influences NFκB, which enhances the immunosuppressive role of MDSCs by upregulating Notch ligand Jagged1‐2 [161]. CHOP also regulates MDSC functions, with higher levels in tumor‐infiltrating MDSCs compared with spleen or bone marrow cells, likely due to ROS or peroxynitrite activating the PERK axis. Increased CHOP expression aggregates MDSCs and suppresses T‐cell activity by upregulating IL‐6 and Arg1 [162]. PERK reprograms hemopoietic stem cells in mice spleens into MDSC precursors by activating downstream ATF4 and CCAAT/enhancer‐binding protein beta (C/EBPβ) signals. Targeting PERK prevents myeloid progeny cells from becoming MDSCs even after exposure to the TME, remodeling the TME and inhibiting tumor growth [163]. These findings imply that directly targeting PERK in the spleen during therapy may inhibit the generation of tumor‐promoting microenvironments.

In summary, the activation of UPR pathways drives the conversion of PMNs into PMN‐MDSCs that possess immunosuppressive properties. This finding aligns with previous observations demonstrating that UPR impedes the activity and functionality of various cells involved in antitumor immunity. It raises intriguing questions about why activated UPR pathways suppress antitumor immunity, exerting contrasting effects on different cell types within the TME. Further exploration is necessary to unravel the underlying mechanisms behind this phenomenon.

## 4 ER Stress and Cancer Therapy

Based on the understanding of immune escape mechanisms of cancer, targeted immunotherapies have been developed. The immune system can reactivate or enhance antitumor immunity to inhibit tumor cell immune escape. Several immunotherapeutic strategies have been proposed, including immune checkpoint inhibitors, adoptive cell therapy, oncolytic viruses, and malignant tumor vaccines [164]. While significant clinical effects have been observed in certain cancers like melanoma, non‐small cell lung cancer, and hepatoma, many patients still do not benefit from immunotherapy [165]. Additionally, patients who use immunotherapy may also be susceptible to tumor drug resistance, recurrence [166], or treatment‐related toxicity [164]. The severe ER stress and strong UPR activation in cancer cells and immune cells within the TME play a pivotal role in influencing the inherent antitumor immunity, thereby promoting tumor cell immune escape and progression. Therefore, targeting ER stress‐related factors could enhance immunotherapeutic efficacy and expand the scope of immunotherapy strategies (Table 1).

**TABLE 1:** The summary of different types of drugs targeting unfolded protein response.

Class		Compound	Mechanism	Status	References
GRP78 inhibitors		BMTP78	Coupling csGRP78 binding peptide WIFPWIQL with proapoptotic peptide to promote apoptosis	Preclinical development	[167]
		BC71	Combining with csGRP78 to promote apoptosis	Preclinical development	[168]
		ISM	Combining with csGRP78 to promote apoptosis	Preclinical development	[169]
		Par‐4	Combining with csGRP78 to promote apoptosis	Preclinical development	[170]
		SubAB	Degrading GRP78	Preclinical development	[171]
		YUM70	Changing GRP78 conformation	Preclinical development	[172]
		FL‐5	Combining with csGRP78 to promote apoptosis	Preclinical development	[173]
		GRP78 CAR T cells	Killing GRP78‐positive cell	Preclinical development	[174]
IRE1α inhibitors	IRE1α kinase inhibitor	APY29	Type I IRE1α kinase inhibitor	Preclinical development	[175]
		APY24	Type I IRE1α kinase inhibitor	Preclinical development	[175]
		Sunitinib	Type I IRE1α kinase inhibitor	In clinical use	[175]
		Compound 3	Type II IRE1α kinase inhibitor	Preclinical development	[176]
		KIRA6‐9	Type II IRE1α kinase inhibitor	Preclinical development	[177, 178, 179, 180]
	IRE1α RNase inhibitor	4μ8C	Inhibiting XBP1 splicing	Preclinical development	[181]
		STF‐083010	Inhibiting XBP1 splicing	Preclinical development	[182]
		PAIR	Blocking RIDD but retains XBP1 splicing	Preclinical development	[180]
		MKC8866	Inhibiting XBP1 splicing	Preclinical development	[183]
PERK inhibitors		GSK2656157	ATP‐competitive PERK inhibitor	Preclinical development	[184]
		GSK2606414	ATP‐competitive PERK inhibitor	Preclinical development	[185]
ATF6 inhibitors		Ceapin‐A7	Inhibiting ATF6α	Preclinical development	[186]
		Melatonin	Inhibiting ATF6	Preclinical development	[187]

*Abbreviations*: GRP78, glucose‐regulated protein of 78 kDa; csGRP78, cell surface GRP78; ISM, isthmin; Par‐4, prostate apoptosis response‐4; SubAB, subtilase cytotoxin; IRE1α, inositol‐requiring enzyme 1 α; KIRA6‐9, kinase inhibiting RNase attenuator 6–9; XBP1, X‐box binding protein 1; RIDD, regulated IRE1‐dependent decay; PERK, protein kinase R‐like endoplasmic reticulum resident kinase; ATF6, activating transcription factor 6; RNase: ribonuclease; PAIR: partial antagonists of IRE1α RNase; BMTP78: bone metastasis targeting peptide 78; RNase: ribonuclease.

### 4.1 GRP78 Inhibitors

GRP78, which is overexpressed in various tumor cells, correlates with key processes such as proliferation, apoptosis resistance, angiogenesis, invasion, metastasis, and drug resistance [188]. A portion of the overexpressed GRP78 translocates to the cell surface, playing a role as a signal transducer. Consequently, csGRP78 predominantly exists on tumor cells rather than normal cell surfaces [57]. Identifying compounds that can directly inhibit GRP78 in tumor cells holds promise for regulating ER stress and promoting antitumor immunity. Several compounds have been developed to target GRP78 effectively and demonstrate antitumor properties in both in vivo and in vitro settings.

To target GRP78, specific polypeptide molecules with good selectivity and low cytotoxicity have been designed. One such therapeutic agent is bone metastasis targeting peptide 78 (BMTP78), a therapeutic drug that targets GRP78 [189]. It couples the GRP78 binding peptide with a proapoptotic peptide (WIFPWIQL) [167]. BMTP78 selectively targets csGRP78 on cancer cells, leaving normal cells unaffected due to their low csGRP78 expressions. By disrupting mitochondrion membrane permeability, BMTP78 induces apoptosis in target cells. In addition to inhibiting primary tumor growth, BMTP78 eradicates metastatic cancer cells, preventing cancer cell invasion and significantly prolonging the survival of breast cancer mice with lung metastasis [190]. Another GRP78‐specific cyclic peptide, BC71, was designed within the C‐terminal adhesion‐associated domain of the MUC4 and other proteins (AMOP) domain of the proapoptotic protein isthmin (ISM). BC71 selectively targets csGRP78 in breast cancer and tumor vascular endothelial cells in mice models. It achieves this selectivity by binding to the N‐terminal region of GRP78, which also interacts with adenosine‐triphosphate (ATP). BC71 induces apoptosis in breast cancer cells in a caspase‐8‐dependent manner. Systemic BC71 treatment in breast cancer mice promotes tumor cell apoptosis and significantly suppresses tumor angiogenesis without notable weight loss, or liver or kidney dysfunction in the mice [168].

Some naturally occurring proteins have been found to promote tumor cell apoptosis by binding to GRP78. It has been reported that other proteins can also target GRP78, resulting in targeted apoptosis. As a typical proapoptotic protein, ISM is a high‐affinity ligand for GRP78. The binding of ISM and GRP78 creates a complex that exerts proapoptotic effects in activated endothelial and cancer cells expressing high levels of GRP78, thereby inhibiting tumor progression and angiogenesis. Following the binding of ISM and GRP78, they are internalized through a clathrin‐dependent process and colocalize in the mitochondrion. This interaction disrupts the exchange of cytoplasmic ADP and mitochondrial matrix ATP, as it interferes with the function of the ADP/ATP translocase located on the inner mitochondrial membrane. In an experimental study involving systemic administration of soluble ISM (IL‐12 suppression molecule), it was found that ISM inhibited the growth of melanoma and breast cancer cells, thereby suppressing tumor‐associated angiogenesis. It also decreased in tumor cell abundance and formation of peripheral blood vessels, probably due to the enhanced apoptosis of tumor cells and tumor vascular endothelial cells [169]. Prostate apoptosis response‐4 is also a proapoptotic protein. It combines with GRP78 through its SAC structural domain and activates caspase‐8 and caspase‐3 in a Fas‐associated death domain‐dependent manner to induce specific tumor cells apoptosis [170]. In addition to inducing apoptosis through its interaction with GRP78, subtilase cytotoxin (SubAB) hinders tumor advancement by catalyzing GRP78 hydrolysis. Specifically, SubAB uniquely targets GRP78, cleaving between leucine residues at positions 416 and 417 on the hinge region that links the ATPase and protein‐binding domain [171]. This discovery paves the way for the design of a specialized proteolytic enzyme with the potential to target GRP78 to treat cancer patients.

Besides the above biological factors, other molecules have also known to exert antitumor effects by targeting GRP78. YUM70 is a hydroxyquinoline analog that can selectively interact with the C‐terminal SBD of GRP78, causing conformational changes in GRP78 and inhibiting its enzymatic activity. This also causes a significant increase in the expression of FAM129A in pancreatic cancer cells. When applied alone, YUM70 exerts a moderate efficacy in treating pancreatic cancer, both in vivo and in vitro, without causing cytotoxic effects to normal tissues. Furthermore, YUM70 synergizes when combined with topotecan, vorinostat, and MG132, leading to enhanced tumor cell apoptosis [172]. Similarly, 2‐(4‐((4‐acetamidophenoxy)methyl)phenyl)‐N‐isobutylbenzofuran‐3‐carboxamide (FL‐5) exhibits a high affinity for GRP78. FL‐5 primarily targets csGRP78, exerting minimal influence on its ATPase activities. As a result, it promotes apoptosis in tumor vascular endothelial cells and renal cancer cells, sparing normal cells. This underscores its potential as an anticancer agent and inhibitor of tumor angiogenesis [173].

Chimeric antigen receptor (CAR) T therapy has been reported to effectively treat cancer by selectively targeting GRP78. In a previous study, CAR T cells were engineered to target GRP78, enabling them to recognize and eliminate AML cells and various solid tumors expressing this protein [191]. A stress‐induced mechanism occurs during CAR T cell generation, causing GRP78 to migrate to the cell surfaces briefly [174]. This inadvertently leads to GRP78 CAR T cells attacking one another, reducing their effectiveness and longevity. Fortunately, dasatinib can antagonize this phenomenon. Dasatinib enhances the resilience of these cells by preventing the translocation of GRP78 CAR T cells to the cell surface during the manufacturing of GRP78 CAR T cells [174]. This underscores the importance of identifying specific tumor targets to design CAR T cells capable of distinguishing tumor cells from normal cells, ultimately advancing CAR T cell therapy. The presence of GRP78 on cell surfaces makes it a key target for tumor‐directed therapy. Further research is needed to promote the translation of these laboratory findings into clinical applications.

### 4.2 IRE1α Axis Inhibitor

Activation of the IRE1α axis by ER stress promotes cancer immune escape. IRE1α activates the specific splicing of XBP1 and also causes RIDD. Suppression of the IRE1α kinase structural domain or inhibition of the IRE1α RNase structural domain inhibits the IRE1α axis [192].

IRE1α kinase inhibitors are grouped into Type I and Type II. Although both IRE1α kinase inhibitors can deactivate the trans‐autophosphorylation of IRE1α, they exert opposite effects on RNase activity. Type I IRE1α kinase inhibitors enhance the active conformation of the IRE1α kinase catalytic structural domain, thereby activating its adjacent RNase structural domain through allosteric mechanisms. A prototypical type I IRE1α kinase inhibitor, APY29, interacts with the nucleotide‐binding site in a manner akin to the binding between the nucleotide‐binding site and ADP. This interaction stabilizes the conformation of the ATP‐binding site, leading to the activation of RNase [175]. Although activation of RNase induces the splicing of XBP1, overactivation of RNase promotes the occurrence of RIDD, which induces apoptosis in the context of long‐term and severe ER stress [193].

Type II IRE1α kinase inhibitors can stabilize the inactive ATP binding site conformation of IRE1α and inhibit RNase. Compound 3 is the first type II IRE1α kinase inhibitor to be discovered and the only compound that can decrease the activities of IRE1α kinases in vivo and in vitro. Similar to APY29, it reduces the autophosphorylation of IRE1α kinases in a dose‐dependent manner in vitro. Compound 3 can inhibit the splicing of XBP1 mRNA, both in vivo and in vitro, even under ER stress conditions [176]. This indicates that Compound 3 is a KIRA. KIRAs stabilize the conformation of the DFG‐out kinase structural domain and induce a shift in helix‐αC, thereby preventing the formation of back‐to‐back dimers. Consequently, both kinase and RNase activities are suppressed [194]. Building on this foundation, several compounds, such as KIRA6 [177], KIRA7 [178], KIRA8 [179], and KIRA9 [180], have been developed. These chemicals can target IRE1α in both in vivo and in vitro environments, maintaining cellular and tissue viability under ER stress. KIRA6 and KIRA8 can inhibit premature autoimmune diabetes‐induced degeneration of pancreatic islet β cells under ER stress by inhibiting the IRE1α axis [179]. Injection of KIRA6 into the vitreous fluid of rats exposed to ER stress prevented the reduction in the number and functions of photoreceptor cells [177]. KIRA7 and KIRA8 prevent or reduce bleomycin‐induced lung fibrosis and significantly downregulate the mRNA levels of collagen 1A1 and fibronectin [178]. In cancer, KIRA8 significantly inhibited the growth of multiple myeloma but had no effects on normal cells at the dose that inhibited tumor growth [195].

Several forms of IRE1α RNase inhibitors have been created, including numerous small molecular compounds such as 4μ8C [181], STF‐083010 [182], partial antagonists of IRE1α RNase (PAIR) [180], and MKC8866 [183]. These inhibitors can directly inhibit IRE1α RNase activities in a concentration‐dependent manner without altering kinase activities, thereby suppressing XBP1 splicing. STF‐083010 has demonstrated the ability to suppress ER stress‐induced XBP1 splicing under diverse circumstances, including tunicamycin therapy, severe hypoxia, and glucose deprivation, exhibiting time‐ and dose‐dependent selective lethal effects on multiple myeloma cells both in vivo and in vitro [182]. MKC8866 is a salicylaldehyde analogue that has been proven to inhibit the activity of IRE1α to reduce the secretion of various tumor‐promoting factors, including IL‐6, IL‐8, chemokine ligand 1 (CXCL1), transforming growth factor β2 (TGFβ2), and GM‐CSF, to inhibit breast cancer cell proliferation. Various chemotherapeutic agents, including taxol, can increase the activity of IRE1α RNase in breast cancer cells. However, when combined with MKC8866, this effect is mitigated, improving taxol's in vivo efficacy [196]. MKC8866 has also shown promise in enhancing the effectiveness of existing clinical drugs in prostate cancer [197], ovarian cancer [198], and glioblastoma [199]. In contrast to other IRE1α RNase inhibitors that inhibit both XBP1 splicing and RIDD, PAIR represents a novel inhibitor that selectively hinders IRE1α RNase. It displaces helix‐αC in the IRE1α kinase structural domain from its active conformation. Thus, although PAIR facilitates XBP1 splicing, it effectively prevents the initiation of RIDD. This unique mechanism positions PAIR as a potential therapeutic candidate for the treatment of critical diseases characterized by retained XBP1 splicing.

### 4.3 PERK Axis Inhibitor

As previously stated, PERK enhances the expression of ATF4 and CHOP, facilitating growth, progression, and immune evasion in cancer under ER stress. Therefore, inhibition of PERK may have therapeutic effects on cancer. GSK2656157 and GSK2606414 are typical ATP‐competitive PERK inhibitors with a high selectivity for PERK [200]. Their antitumor effects have been demonstrated in various models. In various pancreatic cancer cell lines, pretreating cells with GSK2656157 led to a notable suppression of PERK activation under ER stress conditions. This treatment also decreased the expression of downstream products including ATF4, CHOP, and phosphorylated eIF2α [184]. Moreover, in mouse models, GSK2656157 exhibited significant inhibitory effects on the progression of pancreatic cancer and multiple myeloma. Additionally, it disrupted amino acid metabolism and reduced tumor vascular density [184]. Another noteworthy compound, GSK2606414, is an orally administered selective PERK inhibitor [185]. Studies have demonstrated its effectiveness in inhibiting BZW1‐mediated activation of PERK and eIF2α, along with their phosphorylation in pancreatic ductal carcinoma. This led to a substantial suppression of tumor growth and an extension in the survival time of the mouse models [201]. Meanwhile, GSK2606414 can inhibit the PERK–eIF2α–ERK1/2 axis, thereby suppressing tumor angiogenesis, growth and metastasis [202]. In multiple myeloma, application of GSK2606414 alone or in combination with bortezomib significantly inhibited cancer proliferation and promoted apoptosis [203]. While it has been seen that blocking PERK can slow down the growth of tumors, blocking it has also been linked to cytotoxic effects on exocrine and pancreatic β cells from dogs, rats, and mice. These effects lead to the disruption of glucose homeostasis and an acute attack of insulin‐dependent diabetes [204]. Therefore, developing PERK inhibitors that can selectively target tumor cells while avoiding pancreatic toxicity is vital.

### 4.4 ATF6 Inhibitor

Although the role of ATF6 in cancer immune escape mechanisms has not been fully elucidated, several studies have shown that it regulates tumor occurrence and progression. Activation of ATF6 contributes to the occurrence of colorectal cancer [205] and hepatoma [206] and enhances tumor angiogenesis via the ATF6–EGF pathway [207]. It has also been demonstrated that ATF6 regulates the survival of dormant tumor cells in the human body. Therefore, the inhibition of ATF6 may have immunotherapeutic effects.

Ceapins, a group of pyrazole amides, exhibit selective inhibition of ATF6, with Ceapin‐A7 having the most pronounced effects. Ceapin‐A7 selectively inhibits ATF6α, without affecting the PERK and IRE1α pathways. Furthermore, it does not interfere with the processing of ATF6β or SREBPs, which is jointly mediated by S1P and S2P. Moreover, Ceapins do not induce cytotoxic effects on non‐ER stressed cells [186]. This suggests that further optimization of Ceapins, whether used individually or in combination with existing drugs may provide effective tumor immunotherapy strategies.

Unlike Ceapins, which are synthetic, melatonin is an endogenous hormone that is secreted by the pineal gland. It exhibits significant antioxidant and anti‐inflammatory properties and regulates the sleep‐wake cycle. It has been shown to selectively inhibit ATF6 thereby downregulate ATF6α expression, decrease cyclooxygenase‐2 (COX‐2) levels, and promote apoptosis of hepatoma cells under ER stress [187]. Additionally, melatonin can protect normal cells and reduce radiotherapy‐induced damage to such cells by reducing oxidative stress and DNA damage [208]. While the inhibition of ATF6α activation is an established mechanism through which Melatonin hinders tumorigenesis and tumor progression [209], it is important to note that Melatonin's antitumor effects have been substantiated in various studies [210]. Consequently, further research is warranted to comprehensively assess Melatonin's impacts on tumor treatment, particularly in conjunction with adjuvant radiotherapy, and to explore its potential for clinical applications.

## 5 Clinical Implications and Future Directions

### 5.1 UPR Related Proteins have the Potential to Become Tumor Biomarkers

The role of UPR in tumors has attracted increasing attention. As we have described in the previous article, the UPR signaling pathway is closely related to tumor proliferation, metastasis, and immune escape, thereby affecting tumor progression and prognosis. These mechanisms provide support for the application of UPR as a tumor biomarker. At the same time, the protease fragment of GRP78 was found to be detectable in the serum of HCC patients [211]. This suggests that UPR‐related proteins may be secreted during tumor progression and become potential serum markers for screening or follow‐up of tumor patients. In addition, UPR‐related proteins also have great potential to become biomarkers related to the prognosis of tumor patients. UPR is significantly correlated with the clinical prognosis of HCC patients and can better predict the OS of patients [212, 213]. In addition to the potential to become a marker itself, UPR‐related proteins can also regulate each other with existing tumor markers. IRE1α was found to cleave consensus sequences on genes encoding tumor markers including AFP, PSA, CEA, TG, CA15‐3, and CA125 [214, 215]. CD147 was identified as an inducer of UPR in HCC [216]. This suggests that codetection of tumor markers and UPR‐related proteins may be a better way to monitor tumor progression.

### 5.2 Clinical Trials Targeting UPR

Although many molecules or drugs targeting the UPR signaling pathway have been discovered, they have all remained in the laboratory stage and have not entered clinical trials. Despite this, there are still a small number of clinical trials focusing on the UPR pathway, with targeting GRP78 being the main direction. The anti‐GRP78 monoclonal immunoglobulin M antibody PAT‐SM6 has completed Phase I trials for multiple myeloma (NCT01727778) and melanoma (ACTRN12610000733077) and showed good safety and tolerability [217]. CAR‐T cells targeting GRP78 are also being developed for the treatment of Refractory/Relapsed Hematology and Oncology Disease in Children (ChiCTR2500098785).

### 5.3 CRISPR‐Based Technology and Nanotechnology‐Based Targeted Delivery of UPR Modulators

With advancements in science and technology, the development of CRISPR technology and nanomaterials has enabled precise and efficient targeting of tumor cells. The integration of these technologies with the UPR has introduced a novel approach for cancer treatment. Intratumoral administration of nanoparticle‐based CRISPR/Cas9 targeting Canopy homolog 2 (CNPY2), a critical UPR promoter, has demonstrated significant antitumor effects in HCC tumors [218]. Additionally, polydopamine‐coupled magnetite nanoparticles delivering siRNA against PERK effectively downregulate PERK expression in macrophages derived from mouse peritoneal exudate, promoting their polarization to the M1 phenotype [219]. It is worth noting that nanomaterials themselves can also cause ER stress and UPR activation [220], but this has no obvious damage to cell viability and is selective for tumor cells [221, 222].

## 6 Conclusions

The onset and progression of cancer result from the interplay of numerous complex factors, with the UPR influencing tumor progression through multifaceted mechanisms rather than a singular effect. UPR facilitates tumor angiogenesis, aiding in the clearance of metabolic waste and the delivery of nutrients and oxygen, thereby providing the necessary resources for tumor cell progression and a conduit for metastasis. Furthermore, UPR influences multiple signaling pathways to directly support tumor cell survival and proliferation, while also promoting EMT, which facilitates tumor cell metastasis. Concurrently, within the TME, UPR affects tumor cells by reducing TA–MHC complexes, thereby impeding antigen presentation by APCs and recognition by T cells. In immune cells, UPR suppresses the antitumor activities of NK cells, DCs, and T cells, while simultaneously enhancing the immunosuppressive capabilities of M2 macrophages and MDSCs. Collectively, these factors contribute to the establishment of an immunosuppressive microenvironment conducive to tumor cell survival. Based on the above reasons, ER stress and UPR activation have the potential to serve as new tumor markers.

Focusing on the UPR has become a validated approach for developing new drugs for tumor treatment. Targeted inhibition of GRP78, IRE1α, PERK, and ATF6 can effectively enhance the efficacy of existing cancer treatments and significantly promote cancer cell apoptosis. Currently, several drugs targeting the UPR pathway have been developed with high specificity and low cytotoxicity. These drugs can induce tumor cell death and inhibit cell growth, which contradicts the idea that alleviating ER stress might reestablish cellular homeostasis. Indeed, activation of the UPR in tumor cells and TIICs is a long‐term adaptation to the TME. Since currently developed drugs mainly affect cells with high expression of UPR‐related molecules, the function of TIIC may also be affected. We hypothesize that these UPR‐targeting drugs induce apoptosis by inhibiting tumor cell adaptation to the TME and may also restore the anticancer efficacy of the immune system. The impact of UPR‐targeting drugs on TIIC is unclear. It is necessary to examine whether these drugs affect the survival of TIICs, as they may induce tumor cell death through UPR inhibition. It is important to acknowledge that the UPR encompasses various biological processes and is intricately linked to numerous diseases, necessitating precise delivery of UPR‐targeted therapeutics. Moreover, given that varying intensities and durations of UPR can result in divergent cellular outcomes, careful consideration of drug dosage and the extent of UPR inhibition or activation is crucial in clinical applications. With the development of science and technology, the use of CRISPR‐based technology and nanotechnology‐based targeted delivery has made it possible to use UPR targeted drugs in a precise and personalized way. Further research in these areas will enhance the chances that therapies targeting the UPR will successfully progress through clinical trials and contribute to patient treatment.

Although there has been much research surrounding UPR and tumor immune evasion and treatment, many issues still need to be resolved. All research on UPR‐targeted drugs is still in the laboratory stage, and no preclinical animal experiments or clinical trials have been conducted. Currently, available drugs targeting the UPR mainly target tumor cells and do not consider the impact on immune cells in the TME. At the same time, some drugs are still cytotoxic and can lead to the occurrence of new diseases. Therefore, further research is needed on strategies to restore ER homeostasis in immune cells to enhance immune responses. In addition, priority must be given to improving the selectivity of drugs toward cancer cells while minimizing toxic effects on normal cells. These areas require dedicated attention and research to advance our understanding and pave the way for developing effective treatment strategies. In the future, targeted UPR therapies are expected to become a powerful and effective antitumor preventive strategy, either alone or in combination with existing approaches.

## Author Contributions

R.X.Z., W.L.W., H.J., and X.Y.L. designed this study. R.X.Z. and W.L.W. collected related articles. R.X.Z., W.L.W., B.Z.L., Z.L., and H.J. wrote the manuscript and completed the figures. R.X.Z., W.L.W., B.Z.L., Z.L., H.J., and X.Y.L. revised manuscripts and completed tables. X.Y.L. and H.J. provided funding support. All authors reviewed and approved the final manuscript.

## Ethics Statement

The authors have nothing to report.

## Conflicts of Interest

The authors declare no conflicts of interest.

## Data Availability

The authors have nothing to report.

## References

[1] D. S. Schwarz and M. D. Blower , “The Endoplasmic Reticulum: Structure, Function and Response to Cellular Signaling,” Cellular and Molecular Life Sciences 73, no. 1 (2016): 79–94. 26433683 10.1007/s00018-015-2052-6 PMC4700099

[2] B. M. Adams , M. E. Oster , and D. N. Hebert , “Protein Quality Control in the Endoplasmic Reticulum,” Protein Journal 38, no. 3 (2019): 317–329. 31004255 10.1007/s10930-019-09831-w PMC6589386

[3] S. A. Oakes and F. R. Papa , “The Role of Endoplasmic Reticulum Stress in human Pathology,” Annu Rev Pathol 10 (2015): 173–194. 25387057 10.1146/annurev-pathol-012513-104649 PMC5568783

[4] R. Iurlaro and C. Muñoz‐Pinedo , “Cell Death Induced by Endoplasmic Reticulum Stress,” Febs Journal 283, no. 14 (2016): 2640–2652. 26587781 10.1111/febs.13598

[5] M. Song and J. R. Cubillos‐Ruiz , “Endoplasmic Reticulum Stress Responses in Intratumoral Immune Cells: Implications for Cancer Immunotherapy,” Trends in Immunology 40, no. 2 (2019): 128–141. 30612925 10.1016/j.it.2018.12.001

[6] Z. H. Cao , Z. Wu , C. Hu , M. Zhang , W. Z. Wang , and X. B. Hu , “Endoplasmic Reticulum Stress and Destruction of Pancreatic β Cells in Type 1 Diabetes,” Chinese Medical Journal 133, no. 1 (2020): 68–73. 31923106 10.1097/CM9.0000000000000583 PMC7028193

[7] Q. Liu , H. Körner , H. Wu , and W. Wei , “Endoplasmic Reticulum Stress in Autoimmune Diseases,” Immunobiology 225, no. 2 (2020): 151881. 31879042 10.1016/j.imbio.2019.11.016

[8] C. N. Young , “Endoplasmic Reticulum Stress in the Pathogenesis of Hypertension,” Experimental Physiology 102, no. 8 (2017): 869–884. 28605068 10.1113/EP086274

[9] F. Bray , M. Laversanne , E. Weiderpass , and I. Soerjomataram , “The Ever‐increasing Importance of Cancer as a Leading Cause of Premature Death Worldwide,” Cancer 127, no. 16 (2021): 3029–3030. 34086348 10.1002/cncr.33587

[10] F. Bray , J. Ferlay , I. Soerjomataram , R. L. Siegel , L. A. Torre , and A. Jemal , “Global Cancer Statistics 2018: GLOBOCAN Estimates of Incidence and Mortality Worldwide for 36 Cancers in 185 Countries,” CA: A Cancer Journal for Clinicians 68, no. 6 (2018): 394–424. 30207593 10.3322/caac.21492

[11] F. Bray , M. Laversanne , H. Sung , et al., “Global Cancer Statistics 2022: GLOBOCAN Estimates of Incidence and Mortality Worldwide for 36 Cancers in 185 Countries,” CA: A Cancer Journal for Clinicians 74, no. 3 (2024): 229–263. 38572751 10.3322/caac.21834

[12] S. Chen , Z. Cao , K. Prettner , et al., “Estimates and Projections of the Global Economic Cost of 29 Cancers in 204 Countries and Territories from 2020 to 2050,” JAMA Oncology 9, no. 4 (2023): 465. 36821107 10.1001/jamaoncol.2022.7826 PMC9951101

[13] C. Kim and B. Kim , “Anti‐Cancer Natural Products and Their Bioactive Compounds Inducing ER Stress‐Mediated Apoptosis: A Review,” Nutrients 10, no. 8 (2018): 1021. 30081573 10.3390/nu10081021 PMC6115829

[14] X. Chen , D. Iliopoulos , Q. Zhang , et al., “XBP1 promotes Triple‐negative Breast Cancer by Controlling the HIF1α Pathway,” Nature 508, no. 7494 (2014): 103–107. 24670641 10.1038/nature13119 PMC4105133

[15] L. He , H. Li , C. Li , et al., “HMMR Alleviates Endoplasmic Reticulum Stress by Promoting Autophagolysosomal Activity During Endoplasmic Reticulum Stress‐driven Hepatocellular Carcinoma Progression,” Cancer Commun (Lond) 43, no. 9 (2023): 981–1002. 37405956 10.1002/cac2.12464 PMC10508155

[16] P. Dauer , N. S. Sharma , V. K. Gupta , et al., “ER Stress Sensor, Glucose Regulatory Protein 78 (GRP78) Regulates Redox Status in Pancreatic Cancer Thereby Maintaining “Stemness”,” Cell death & disease 10, no. 2 (2019): 132. 30755605 10.1038/s41419-019-1408-5 PMC6372649

[17] M. M. Chen , W. Guo , S. M. Chen , et al., “Xanthine Dehydrogenase Rewires Metabolism and the Survival of Nutrient Deprived Lung Adenocarcinoma Cells by Facilitating UPR and Autophagic Degradation,” Int J Biol Sci 19, no. 3 (2023): 772–788. 36778128 10.7150/ijbs.78948 PMC9909990

[18] I. C. Salaroglio , E. Panada , E. Moiso , et al., “PERK Induces Resistance to Cell Death Elicited by Endoplasmic Reticulum Stress and Chemotherapy,” Molecular cancer 16, no. 1 (2017): 91. 28499449 10.1186/s12943-017-0657-0 PMC5427528

[19] C. Hetz , K. Zhang , and R. J. Kaufman , “Mechanisms, Regulation and Functions of the Unfolded Protein Response,” Nature Reviews Molecular Cell Biology 21, no. 8 (2020): 421–438. 32457508 10.1038/s41580-020-0250-z PMC8867924

[20] O. Morana , W. Wood , and C. D. Gregory , “The Apoptosis Paradox in Cancer,” International Journal of Molecular Sciences 23, no. 3 (2022): 1328. 35163253 10.3390/ijms23031328 PMC8836235

[21] X. Yi , H. Wang , Y. Yang , et al., “SIRT7 orchestrates Melanoma Progression by Simultaneously Promoting Cell Survival and Immune Evasion via UPR Activation,” Signal Transduct Target Ther 8, no. 1 (2023): 107. 36918544 10.1038/s41392-023-01314-w PMC10015075

[22] W. Y. Xie , X. D. Zhou , Q. Li , L. X. Chen , and D. H. Ran , “Acid‐induced Autophagy Protects human Lung Cancer Cells From Apoptosis by Activating ER Stress,” Experimental Cell Research 339, no. 2 (2015): 270–279. 26559141 10.1016/j.yexcr.2015.11.005

[23] E. Andreucci , S. Peppicelli , J. Ruzzolini , F. Bianchini , and L. Calorini , “Physicochemical Aspects of the Tumour Microenvironment as Drivers of Vasculogenic Mimicry,” Cancer and Metastasis Reviews 41, no. 4 (2022): 935–951. 36224457 10.1007/s10555-022-10067-x PMC9758104

[24] M. Corazzari , F. Rapino , F. Ciccosanti , et al., “Oncogenic BRAF Induces Chronic ER Stress Condition Resulting in Increased Basal Autophagy and Apoptotic Resistance of Cutaneous Melanoma,” Cell Death and Differentiation 22, no. 6 (2015): 946–958. 25361077 10.1038/cdd.2014.183 PMC4423179

[25] A. Goenka , F. Khan , B. Verma , et al., “Tumor Microenvironment Signaling and Therapeutics in Cancer Progression,” Cancer Commun (Lond) 43, no. 5 (2023): 525–561. 37005490 10.1002/cac2.12416 PMC10174093

[26] J. Hwang and L. Qi , “Quality Control in the Endoplasmic Reticulum: Crosstalk Between ERAD and UPR Pathways,” Trends in Biochemical Sciences 43, no. 8 (2018): 593–605. 30056836 10.1016/j.tibs.2018.06.005 PMC6327314

[27] S. X. Zhang , J. J. Wang , C. R. Starr , et al., “The Endoplasmic Reticulum: Homeostasis and Crosstalk in Retinal Health and Disease,” Progress in Retinal and Eye Research 98 (2024): 101231. 38092262 10.1016/j.preteyeres.2023.101231 PMC11056313

[28] Y. Zhang , Y. Wang , G. Zhao , E. J. Tanner , M. Adli , and D. Matei , “FOXK2 promotes Ovarian Cancer Stemness by Regulating the Unfolded Protein Response Pathway,” Journal of Clinical Investigation 132, no. 10 (2022). 10.1172/JCI151591 PMC9106354 35349489

[29] S. Chen , A. Henderson , M. C. Petriello , et al., “Trimethylamine N‐Oxide Binds and Activates PERK to Promote Metabolic Dysfunction,” Cell metabolism 30, no. 6 (2019): 1141–1151.e5. e5. 31543404 10.1016/j.cmet.2019.08.021

[30] C. Hetz , “The Unfolded Protein Response: Controlling Cell Fate Decisions Under ER Stress and Beyond,” Nature Reviews Molecular Cell Biology 13, no. 2 (2012): 89–102. 22251901 10.1038/nrm3270

[31] J. J. Rodvold , N. R. Mahadevan , and M. Zanetti , “Immune Modulation by ER Stress and Inflammation in the Tumor Microenvironment,” Cancer Letters 380, no. 1 (2016): 227–236. 26525580 10.1016/j.canlet.2015.09.009

[32] K. Mori , “Evolutionary Aspects of the Unfolded Protein Response,” Cold Spring Harbor perspectives in biology 14, no. 12 (2022). 10.1101/cshperspect.a041262 PMC9732898 35940910

[33] P. Kettel , L. Marosits , E. Spinetti , et al., “Disordered Regions in the IRE1α ER Lumenal Domain Mediate Its Stress‐induced Clustering,” Embo Journal 43 (2024): 4668–4698. 39232130 10.1038/s44318-024-00207-0 PMC11480506

[34] X. H. Fun and G. Thibault , “Lipid Bilayer Stress and Proteotoxic Stress‐induced Unfolded Protein Response Deploy Divergent Transcriptional and Non‐transcriptional Programmes,” Biochim Biophys Acta Mol Cell Biol Lipids 1865, no. 1 (2020): 158449. 31028913 10.1016/j.bbalip.2019.04.009

[35] B. M. Gardner and P. Walter , “Unfolded Proteins Are Ire1‐activating Ligands That Directly Induce the Unfolded Protein Response,” Science 333, no. 6051 (2011): 1891–1894. 21852455 10.1126/science.1209126 PMC3202989

[36] F. Cairrão , C. C. Santos , A. Le Thomas , S. Marsters , A. Ashkenazi , and P. M. Domingos , “Pumilio Protects Xbp1 mRNA From Regulated Ire1‐dependent Decay,” Nature Communications 13, no. 1 (2022): 1587. 10.1038/s41467-022-29105-x PMC8948244 35332141

[37] L. H. Glimcher , A. H. Lee , and N. N. Iwakoshi , “XBP‐1 and the Unfolded Protein Response (UPR),” Nature Immunology 21, no. 9 (2020): 963–965. 32616861 10.1038/s41590-020-0708-3

[38] M. J. Grey , E. Cloots , M. S. Simpson , et al., “IRE1β negatively Regulates IRE1α Signaling in Response to Endoplasmic Reticulum Stress,” Journal of Cell Biology 219, no. 2 (2020). 10.1083/jcb.201904048 PMC7041686 31985747

[39] E. Cloots , M. S. Simpson , C. De Nolf , W. I. Lencer , S. Janssens , and M. J. Grey , “Evolution and Function of the Epithelial Cell‐specific ER Stress Sensor IRE1β,” Mucosal Immunol 14, no. 6 (2021): 1235–1246. 34075183 10.1038/s41385-021-00412-8 PMC8528705

[40] M. J. Grey , H. De Luca , D. V. Ward , et al., “The Epithelial‐specific ER Stress Sensor ERN2/IRE1β Enables Host‐microbiota Crosstalk to Affect Colon Goblet Cell Development,” Journal of Clinical Investigation 132, no. 17 (2022). 10.1172/JCI153519 PMC9435652 35727638

[41] S. E. Bettigole and L. H. Glimcher , “Endoplasmic Reticulum Stress in Immunity,” Annual Review of Immunology 33 (2015): 107–138. 10.1146/annurev-immunol-032414-112116 25493331

[42] W. Rozpedek , D. Pytel , B. Mucha , H. Leszczynska , J. A. Diehl , and I. Majsterek , “The Role of the PERK/eIF2α/ATF4/CHOP Signaling Pathway in Tumor Progression during Endoplasmic Reticulum Stress,” Current Molecular Medicine 16, no. 6 (2016): 533–544. 27211800 10.2174/1566524016666160523143937 PMC5008685

[43] A. De Leo , A. Ugolini , X. Yu , et al., “Glucose‐driven Histone Lactylation Promotes the Immunosuppressive Activity of Monocyte‐derived Macrophages in Glioblastoma,” Immunity 57, no. 5 (2024): 1105–1123.e8. e8. 38703775 10.1016/j.immuni.2024.04.006 PMC11114377

[44] C. She , C. Wu , W. Guo , et al., “Combination of RUNX1 Inhibitor and Gemcitabine Mitigates Chemo‐resistance in Pancreatic Ductal Adenocarcinoma by Modulating BiP/PERK/eIF2α‐axis‐mediated Endoplasmic Reticulum Stress,” Journal of Experimental & Clinical Cancer Research 42, no. 1 (2023): 238. 37697370 10.1186/s13046-023-02814-x PMC10494371

[45] D. Li , W. J. Wang , Y. Z. Wang , Y. B. Wang , and Y. L. Li , “Lobaplatin Promotes (125)I‐induced Apoptosis and Inhibition of Proliferation in Hepatocellular Carcinoma by Upregulating PERK‐eIF2α‐ATF4‐CHOP Pathway,” Cell death & disease 10, no. 10 (2019): 744. 31582720 10.1038/s41419-019-1918-1 PMC6776519

[46] P. Wang , L. Han , M. Yu , et al., “The Prognostic Value of PERK in Cancer and Its Relationship with Immune Cell Infiltration,” Frontiers in Molecular Biosciences 8 (2021): 648752. 33937330 10.3389/fmolb.2021.648752 PMC8085429

[47] K. Haze , T. Okada , H. Yoshida , et al., “Identification of the G13 (cAMP‐response‐element‐binding protein‐related protein) Gene Product Related to Activating Transcription Factor 6 as a Transcriptional Activator of the Mammalian Unfolded Protein Response,” Biochemical Journal 355, no. Pt 1 (2001): 19–28. 11256944 10.1042/0264-6021:3550019 PMC1221707

[48] W. T. Stauffer , A. Arrieta , E. A. Blackwood , and C. C. Glembotski , “Sledgehammer to Scalpel: Broad Challenges to the Heart and Other Tissues Yield Specific Cellular Responses via Transcriptional Regulation of the ER‐Stress Master Regulator ATF6α,” International Journal of Molecular Sciences 21, no. 3 (2020): 1134. 32046286 10.3390/ijms21031134 PMC7037772

[49] A. Papaioannou , A. Higa , G. Jégou , et al., “Alterations of EDEM1 Functions Enhance ATF6 Pro‐survival Signaling,” Febs Journal 285, no. 22 (2018): 4146–4164. 30281916 10.1111/febs.14669

[50] F. Hinte , E. van Anken , B. Tirosh , and W. Brune , “Repression of Viral Gene Expression and Replication by the Unfolded Protein Response Effector XBP1u,” Elife 9 (2020). 10.7554/eLife.51804 PMC7082126 32065579

[51] Y. Liu , X. Wang , Z. Zhen , Y. Yu , Y. Qiu , and W. Xiang , “GRP78 regulates Milk Biosynthesis and the Proliferation of Bovinemammaryepithelial Cells Through the mTOR Signaling Pathway,” Cellular & Molecular Biology Letters 24 (2019): 57. 31660059 10.1186/s11658-019-0181-x PMC6805561

[52] N. Amin‐Wetzel , R. A. Saunders , M. J. Kamphuis , et al., “A J‐Protein Co‐chaperone Recruits BiP to Monomerize IRE1 and Repress the Unfolded Protein Response,” Cell 171, no. 7 (2017): 1625–1637.e13. e13. 29198525 10.1016/j.cell.2017.10.040 PMC5733394

[53] C. J. Adams , M. C. Kopp , N. Larburu , P. R. Nowak , and M. M. U. Ali , “Structure and Molecular Mechanism of ER Stress Signaling by the Unfolded Protein Response Signal Activator IRE1,” Frontiers in Molecular Biosciences 6 (2019): 11. 30931312 10.3389/fmolb.2019.00011 PMC6423427

[54] M. C. Kopp , N. Larburu , V. Durairaj , C. J. Adams , and M. M. U. Ali , “UPR Proteins IRE1 and PERK Switch BiP From Chaperone to ER Stress Sensor,” Nature structural & molecular biology 26, no. 11 (2019): 1053–1062. 10.1038/s41594-019-0324-9 PMC6858872 31695187

[55] J. Shen , E. L. Snapp , J. Lippincott‐Schwartz , and R. Prywes , “Stable Binding of ATF6 to BiP in the Endoplasmic Reticulum Stress Response,” Molecular and Cellular Biology 25, no. 3 (2005): 921–932. 15657421 10.1128/MCB.25.3.921-932.2005 PMC543992

[56] J. Jia , L. Zhu , X. Yue , et al., “Crosstalk Between KDEL Receptor and EGF Receptor Mediates Cell Proliferation and Migration via STAT3 Signaling,” Cell Communication and Signaling 22, no. 1 (2024): 140. 38378560 10.1186/s12964-024-01517-w PMC10880305

[57] M. Farshbaf , A. Y. Khosroushahi , S. Mojarad‐Jabali , A. Zarebkohan , H. Valizadeh , and P. R. Walker , “Cell Surface GRP78: An Emerging Imaging Marker and Therapeutic Target for Cancer,” J Control Release 328 (2020): 932–941. 33129921 10.1016/j.jconrel.2020.10.055

[58] I. Hernandez and M. Cohen , “Linking Cell‐surface GRP78 to Cancer: From Basic Research to Clinical Value of GRP78 Antibodies,” Cancer Letters 524 (2022): 1–14. 34637844 10.1016/j.canlet.2021.10.004

[59] X. Zeng , H. Zhang , J. Guo , et al., “A Novel Bispecific T‐cell Engager Using the Ligand‐target csGRP78 Against Acute Myeloid Leukemia,” Cellular and Molecular Life Sciences 81, no. 1 (2024): 371. 39196413 10.1007/s00018-024-05410-0 PMC11358366

[60] A. M. Shields , S. J. Thompson , G. S. Panayi , and V. M. Corrigall , “Pro‐resolution Immunological Networks: Binding Immunoglobulin Protein and Other Resolution‐associated Molecular Patterns,” Rheumatology 51, no. 5 (2012): 780–788. 22190690 10.1093/rheumatology/ker412

[61] Z. Wu , Z. Xu , X. Zhou , et al., “sGRP78 enhances Selective Autophagy of Monomeric TLR4 to Regulate Myeloid Cell Death,” Cell death & disease 13, no. 7 (2022): 587. 35798718 10.1038/s41419-022-05048-5 PMC9262968

[62] Y. Tang , Q. Jiang , Y. Ou , et al., “BIP Induces Mice CD19(hi) Regulatory B Cells Producing IL‐10 and Highly Expressing PD‐L1, FasL,” Molecular Immunology 69 (2016): 44–51. 26655428 10.1016/j.molimm.2015.10.017

[63] M. Yang , F. Zhang , K. Qin , et al., “Glucose‐Regulated Protein 78‐Induced Myeloid Antigen‐Presenting Cells Maintained Tolerogenic Signature Upon LPS Stimulation,” Frontiers in immunology 7 (2016): 552. 27990144 10.3389/fimmu.2016.00552 PMC5131008

[64] K. Qin , S. Ma , H. Li , et al., “GRP78 Impairs Production of Lipopolysaccharide‐Induced Cytokines by Interaction With CD14,” Frontiers in immunology 8 (2017): 579. 28588578 10.3389/fimmu.2017.00579 PMC5440525

[65] L. Zhao , Y. Lv , X. Zhou , et al., “Secreted Glucose Regulated protein78 Ameliorates DSS‐induced Mouse Colitis,” Frontiers in immunology 14 (2023): 986175. 36776831 10.3389/fimmu.2023.986175 PMC9909966

[66] L. Chen , H. Zheng , X. Yu , et al., “Tumor‐Secreted GRP78 Promotes the Establishment of a Pre‐metastatic Niche in the Liver Microenvironment,” Frontiers in immunology 11 (2020): 584458. 33133103 10.3389/fimmu.2020.584458 PMC7550426

[67] S. Mafi , E. Ahmadi , E. Meehan , et al., “The mTOR Signaling Pathway Interacts With the ER Stress Response and the Unfolded Protein Response in Cancer,” Cancer Research 83, no. 15 (2023): 2450–2460. 37195095 10.1158/0008-5472.CAN-22-3032

[68] L. Qin , Z. Wang , L. Tao , and Y. Wang , “ER Stress Negatively Regulates AKT/TSC/mTOR Pathway to Enhance Autophagy,” Autophagy 6, no. 2 (2010): 239–247. 20104019 10.4161/auto.6.2.11062

[69] W. Y. Hung , J. H. Chang , Y. Cheng , et al., “Autophagosome Accumulation‐mediated ATP Energy Deprivation Induced by Penfluridol Triggers Nonapoptotic Cell Death of Lung Cancer via Activating Unfolded Protein Response,” Cell death & disease 10, no. 8 (2019): 538. 31308361 10.1038/s41419-019-1785-9 PMC6629704

[70] A. Grenier , L. Poulain , J. Mondesir , et al., “AMPK‐PERK Axis Represses Oxidative Metabolism and Enhances Apoptotic Priming of Mitochondria in Acute Myeloid Leukemia,” Cell reports 38, no. 1 (2022): 110197. 34986346 10.1016/j.celrep.2021.110197

[71] X. Jiang , B. Zhu , G. Li , et al., “p20BAP31 promotes Cell Apoptosis via Interaction With GRP78 and Activating the PERK Pathway in Colorectal Cancer,” International Journal of Biological Macromolecules 272, no. Pt 2 (2024): 132870. 38844291 10.1016/j.ijbiomac.2024.132870

[72] S. K. Niture and A. K. Jaiswal , “Nrf2‐induced Antiapoptotic Bcl‐xL Protein Enhances Cell Survival and Drug Resistance,” Free Radic Biol Med 57 (2013): 119–131. 23275004 10.1016/j.freeradbiomed.2012.12.014 PMC3606082

[73] J. Li , B. Lee , and A. S. Lee , “Endoplasmic Reticulum Stress‐induced Apoptosis: Multiple Pathways and Activation of p53‐up‐regulated Modulator of Apoptosis (PUMA) and NOXA by p53,” Journal of Biological Chemistry 281, no. 11 (2006): 7260–7270. 16407291 10.1074/jbc.M509868200

[74] Y. Qian , C. C. Wong , J. Xu , et al., “Sodium Channel Subunit SCNN1B Suppresses Gastric Cancer Growth and Metastasis via GRP78 Degradation,” Cancer Research 77, no. 8 (2017): 1968–1982. 28202509 10.1158/0008-5472.CAN-16-1595

[75] S. S. Choi , S. K. Lee , J. K. Kim , et al., “Flightless‐1 Inhibits ER Stress‐induced Apoptosis in Colorectal Cancer Cells by Regulating Ca(2+) Homeostasis,” Experimental & Molecular Medicine 52, no. 6 (2020): 940–950. 32504039 10.1038/s12276-020-0448-3 PMC7338537

[76] Y. X. Feng , E. S. Sokol , C. A. Del Vecchio , et al., “Epithelial‐to‐mesenchymal Transition Activates PERK‐eIF2α and Sensitizes Cells to Endoplasmic Reticulum Stress,” Cancer discovery 4, no. 6 (2014): 702–715. 24705811 10.1158/2159-8290.CD-13-0945

[77] S. Dey , C. M. Sayers , Verginadis II , et al., “ATF4‐dependent Induction of Heme Oxygenase 1 Prevents Anoikis and Promotes Metastasis,” Journal of Clinical Investigation 125, no. 7 (2015): 2592–2608. 26011642 10.1172/JCI78031 PMC4563676

[78] H. Yuan , Z. Zhao , Z. Guo , L. Ma , J. Han , and Y Song . A Novel ER Stress Mediator TMTC3 Promotes Squamous Cell Carcinoma Progression by Activating GRP78/PERK Signaling Pathway. Int J Biol Sci 2022;18(13):4853–4868. 35982901 10.7150/ijbs.72838 PMC9379397

[79] P. Carmeliet and R. K Jain . Molecular Mechanisms and Clinical Applications of Angiogenesis. Nature 2011;473(7347):298–307. 21593862 10.1038/nature10144 PMC4049445

[80] R. Ghosh , K. L. Lipson , K. E. Sargent , et al. Transcriptional Regulation of VEGF‐A by the Unfolded Protein Response Pathway. PLoS ONE 2010;5(3):e9575. 20221394 10.1371/journal.pone.0009575 PMC2833197

[81] E. R. Pereira , N. Liao , G. A. Neale , and L. M Hendershot . Transcriptional and Post‐transcriptional Regulation of Proangiogenic Factors by the Unfolded Protein Response. PLoS ONE e12521, 2010;5(9). 20824063 10.1371/journal.pone.0012521 PMC2932741

[82] G. Auf , A. Jabouille , S. Guérit , et al. Inositol‐requiring Enzyme 1alpha Is a Key Regulator of Angiogenesis and Invasion in Malignant Glioma. PNAS 2010;107(35):15553–15558. 20702765 10.1073/pnas.0914072107 PMC2932600

[83] J. M. Harnoss , A. Le Thomas , and M. Reichelt , et al. IRE1α Disruption in Triple‐Negative Breast Cancer Cooperates With Antiangiogenic Therapy by Reversing ER Stress Adaptation and Remodeling the Tumor Microenvironment. Cancer Research 2020;80(11):2368–2379. 32265225 10.1158/0008-5472.CAN-19-3108 PMC7272310

[84] Y. Wang , G. N. Alam , Y. Ning , et al. The Unfolded Protein Response Induces the Angiogenic Switch in human Tumor Cells Through the PERK/ATF4 Pathway. Cancer Research 2012;72(20):5396–5406. 22915762 10.1158/0008-5472.CAN-12-0474 PMC3743425

[85] M. Peng , Y. Mo , Y. Wang , et al. Neoantigen Vaccine: An Emerging Tumor Immunotherapy. Molecular cancer 2019;18(1):128. 31443694 10.1186/s12943-019-1055-6 PMC6708248

[86] M. W. Teng , J. Galon , W. H. Fridman , and M. J Smyth . From Mice to Humans: Developments in Cancer Immunoediting. Journal of Clinical Investigation 2015;125(9):3338–3346. 26241053 10.1172/JCI80004 PMC4588291

[87] M. M. Gubin and M. D Vesely . Cancer Immunoediting in the Era of Immuno‐oncology. Clinical Cancer Research 28, 3917–3928, 2022. 35594163 10.1158/1078-0432.CCR-21-1804 PMC9481657

[88] G. Kroemer , T. A. Chan , A. M. M. Eggermont , and L Galluzzi . Immunosurveillance in Clinical Cancer Management. CA: A Cancer Journal for Clinicians 2024;74(2):187–202. 37880100 10.3322/caac.21818 PMC10939974

[89] O. Demaria , S. Cornen , M. Daëron , Y. Morel , R. Medzhitov , and E Vivier . Harnessing Innate Immunity in Cancer Therapy. Nature 2019;574(7776):45–56. 31578484 10.1038/s41586-019-1593-5

[90] H. Qin and Y Chen . Lipid Metabolism and Tumor Antigen Presentation. Advances in Experimental Medicine and Biology 2021;1316:169–189. 33740250 10.1007/978-981-33-6785-2_11

[91] S. Jhunjhunwala , C. Hammer , and L Delamarre . Antigen Presentation in Cancer: Insights Into Tumour Immunogenicity and Immune Evasion. Nature Reviews Cancer 2021;21(5):298–312. 33750922 10.1038/s41568-021-00339-z

[92] T. Jiang , T. Shi , H. Zhang , et al. Tumor Neoantigens: From Basic Research to Clinical Applications. Journal of hematology & oncology 2019;12(1):93. 31492199 10.1186/s13045-019-0787-5 PMC6731555

[93] R. Y. Pan , W. H. Chung , M. T. Chu , et al. Recent Development and Clinical Application of Cancer Vaccine: Targeting Neoantigens. Journal of Immunology Research 2018;2018:1–9. 10.1155/2018/4325874 PMC6313977 30662919

[94] F. Lang , B. Schrörs , M. Löwer , Ö. Türeci , and U Sahin . Identification of Neoantigens for Individualized Therapeutic Cancer Vaccines. Nat Rev Drug Discovery 2022;21(4):261–282. 35105974 10.1038/s41573-021-00387-y PMC7612664

[95] T. E. Angell , M. G. Lechner , J. K. Jang , J. S. LoPresti , and A. L Epstein . MHC Class I Loss Is a Frequent Mechanism of Immune Escape in Papillary Thyroid Cancer That Is Reversed by Interferon and Selumetinib Treatment in Vitro. Clinical Cancer Research 2014;20(23):6034–6044. 25294906 10.1158/1078-0432.CCR-14-0879 PMC4252612

[96] F. Perea , M. Bernal , A. Sánchez‐Palencia , et al. The Absence of HLA Class I Expression in Non‐small Cell Lung Cancer Correlates With the Tumor Tissue Structure and the Pattern of T Cell Infiltration. International Journal of Cancer 2017;140(4):888–899. 27785783 10.1002/ijc.30489

[97] M. Kawazu , T. Ueno , K. Saeki , et al. HLA Class I Analysis Provides Insight into the Genetic and Epigenetic Background of Immune Evasion in Colorectal Cancer with High Microsatellite Instability. Gastroenterology 2022;162(3):799–812. 34687740 10.1053/j.gastro.2021.10.010

[98] M. L. Burr , C. E. Sparbier , K. L. Chan , et al. An Evolutionarily Conserved Function of Polycomb Silences the MHC Class I Antigen Presentation Pathway and Enables Immune Evasion in Cancer. Cancer Cell 2019;36(4):385–401.e8.e8. 31564637 10.1016/j.ccell.2019.08.008 PMC6876280

[99] S. F. de Almeida , J. V. Fleming , J. E. Azevedo , M. Carmo‐Fonseca , and M de Sousa . Stimulation of an Unfolded Protein Response Impairs MHC Class I Expression. Journal of Immunology 2007;178(6):3612–3619. 10.4049/jimmunol.178.6.3612 17339458

[100] D. P. Granados , P. L. Tanguay , M. P. Hardy , et al. ER Stress Affects Processing of MHC Class I‐associated Peptides. BMC Immunology [Electronic Resource] 2009;10:10. 19220912 10.1186/1471-2172-10-10 PMC2657905

[101] A. Pommier , N. Anaparthy , N. Memos , et al. Unresolved Endoplasmic Reticulum Stress Engenders Immune‐resistant, Latent Pancreatic Cancer Metastases. Science 2018;360(6394). 10.1126/science.aao4908 PMC6547380 29773669

[102] M. Ganesan , S. Mathews , E. Makarov , et al. Acetaldehyde Suppresses HBV‐MHC Class I Complex Presentation on Hepatocytes via Induction of ER Stress and Golgi Fragmentation. American journal of physiology Gastrointestinal and liver physiology 2020;319(4):G432–G442. 32755306 10.1152/ajpgi.00109.2020 PMC7654643

[103] R. Bartoszewski , J. W. Brewer , and A. Rab , et al. The Unfolded Protein Response (UPR)‐activated Transcription Factor X‐box‐binding Protein 1 (XBP1) Induces microRNA‐346 Expression That Targets the human Antigen Peptide Transporter 1 (TAP1) mRNA and Governs Immune Regulatory Genes. Journal of Biological Chemistry 2011;286(48):41862–41870. 22002058 10.1074/jbc.M111.304956 PMC3308892

[104] E. Vivier , D. Artis , M. Colonna , et al. Innate Lymphoid Cells: 10 Years On. Cell 2018;174(5):1054–1066. 30142344 10.1016/j.cell.2018.07.017

[105] N. D. Huntington , J. Cursons , and J Rautela . The Cancer‐natural Killer Cell Immunity Cycle. Nature Reviews Cancer 2020;20(8):437–454. 32581320 10.1038/s41568-020-0272-z

[106] I. Prager and C Watzl . Mechanisms of Natural Killer Cell‐mediated Cellular Cytotoxicity. J Leukoc Biol 2019;105(6):1319–1329. 31107565 10.1002/JLB.MR0718-269R

[107] C. Guillerey , N. D. Huntington , and M. J Smyth . Targeting Natural Killer Cells in Cancer Immunotherapy. Nature Immunology 2016;17(9):1025–1036. 27540992 10.1038/ni.3518

[108] S. Sarkar , W. T. Germeraad , K. M. Rouschop , et al. Hypoxia Induced Impairment of NK Cell Cytotoxicity Against Multiple Myeloma Can be Overcome by IL‐2 Activation of the NK Cells. PLoS ONE 2013;8(5):e64835. 23724099 10.1371/journal.pone.0064835 PMC3665801

[109] A. Obiedat , E. Seidel , M. Mahameed , et al. Transcription of the NKG2D Ligand MICA Is Suppressed by the IRE1/XBP1 Pathway of the Unfolded Protein Response Through the Regulation of E2F1. Faseb Journal 2019;33(3):3481–3495. 30452881 10.1096/fj.201801350RR

[110] B. G. Gowen , B. Chim , C. D. Marceau , et al. A Forward Genetic Screen Reveals Novel Independent Regulators of ULBP1, an Activating Ligand for Natural Killer Cells. Elife 2015;4. 10.7554/eLife.08474 PMC4629278 26565589

[111] M. Lazarova and A Steinle . The NKG2D Axis: An Emerging Target in Cancer Immunotherapy. Expert Opinion on Therapeutic Targets 2019;23(4):281–294. 30732494 10.1080/14728222.2019.1580693

[112] S. Zhu , N. Yang , J. Wu , et al. Tumor Microenvironment‐related Dendritic Cell Deficiency: A Target to Enhance Tumor Immunotherapy. Pharmacological Research 2020;159:104980. 32504832 10.1016/j.phrs.2020.104980

[113] S. Balan , M. Saxena , and N Bhardwaj . Dendritic Cell Subsets and Locations. Int Rev Cell Mol Biol 2019;348:1–68. 31810551 10.1016/bs.ircmb.2019.07.004

[114] G. J. Clark , P. A. Silveira , P. M. Hogarth , and D. N. J Hart . The Cell Surface Phenotype of human Dendritic Cells. Seminars in cell & developmental biology 2019;86:3–14. 29499385 10.1016/j.semcdb.2018.02.013

[115] A. Lanzavecchia and F Sallusto . Antigen Decoding by T Lymphocytes: From Synapses to Fate Determination. Nature Immunology 2001;2(6):487–492. 11376334 10.1038/88678

[116] C. S. Garris and M. J Pittet . ER Stress in Dendritic Cells Promotes Cancer. Cell 2015;161(7):1492–1493. 26091029 10.1016/j.cell.2015.06.006

[117] J. R. Cubillos‐Ruiz , P. C. Silberman , M. R. Rutkowski , et al. ER Stress Sensor XBP1 Controls Anti‐tumor Immunity by Disrupting Dendritic Cell Homeostasis. Cell 2015;161(7):1527–1538. 26073941 10.1016/j.cell.2015.05.025 PMC4580135

[118] D. L. Herber , W. Cao , Y. Nefedova , et al. Lipid Accumulation and Dendritic Cell Dysfunction in Cancer. Nature Medicine 2010;16(8):880–886. 10.1038/nm.2172 PMC2917488 20622859

[119] M. S. Gilardini Montani , R. Benedetti , and S. Piconese , et al. PGE2 Released by Pancreatic Cancer Cells Undergoing ER Stress Transfers the Stress to DCs Impairing Their Immune Function. Molecular Cancer Therapeutics 2021;20(5):934–945. 33632872 10.1158/1535-7163.MCT-20-0699

[120] O. Guttman , A. Le Thomas , and S. Marsters , et al. Antigen‐derived Peptides Engage the ER Stress Sensor IRE1α to Curb Dendritic Cell Cross‐presentation. Journal of Cell Biology 2022;221(6). 10.1083/jcb.202111068 PMC9036094 35446348

[121] N. R. Mahadevan , V. Anufreichik , J. J. Rodvold , K. T. Chiu , H. Sepulveda , and M Zanetti . Cell‐extrinsic Effects of Tumor ER Stress Imprint Myeloid Dendritic Cells and Impair CD8⁺ T Cell Priming. PLoS ONE 2012;7(12):e51845. 23272178 10.1371/journal.pone.0051845 PMC3525659

[122] Z. Zeng , H. Y. Chew , J. G. Cruz , G. R. Leggatt , and J. W Wells . Investigating T Cell Immunity in Cancer: Achievements and Prospects. International Journal of Molecular Sciences 2907, 2021;22(6). 33809369 10.3390/ijms22062907 PMC7999898

[123] B. Farhood , M. Najafi , and K Mortezaee . CD8(+) cytotoxic T Lymphocytes in Cancer Immunotherapy: A Review. Journal of Cellular Physiology 2019;234(6):8509–8521. 30520029 10.1002/jcp.27782

[124] B. J. Laidlaw , J. E. Craft , and S. M Kaech . The Multifaceted Role of CD4(+) T Cells in CD8(+) T Cell Memory. Nature Reviews Immunology 2016;16(2):102–111. 10.1038/nri.2015.10 PMC4860014 26781939

[125] A. Palazon , P. A. Tyrakis , D. Macias , et al. An HIF‐1α/VEGF‐A Axis in Cytotoxic T Cells Regulates Tumor Progression. Cancer Cell 2017;32(5):669–683.e5.e5. 29136509 10.1016/j.ccell.2017.10.003 PMC5691891

[126] R. Wu and K. M Murphy . DCs at the Center of Help: Origins and Evolution of the Three‐cell‐type Hypothesis. Journal of Experimental Medicine 2022;219(7). 10.1084/jem.20211519 PMC9098650 35543702

[127] S. P. Schoenberger , R. E. Toes , E. I. van der Voort , R. Offringa , and C. J Melief . T‐cell Help for Cytotoxic T Lymphocytes Is Mediated by CD40‐CD40L Interactions. Nature 1998;393(6684):480–483. 9624005 10.1038/31002

[128] S. Feau , Z. Garcia , R. Arens , H. Yagita , J. Borst , and S. P Schoenberger . The CD4⁺ T‐cell Help Signal Is Transmitted From APC to CD8⁺ T‐cells via CD27‐CD70 Interactions. Nature Communications 2012;3:948. 10.1038/ncomms1948 PMC3606886 22781761

[129] K. E. Hurst , K. A. Lawrence , M. T. Essman , Z. J. Walton , L. R. Leddy , and J. E Thaxton . Endoplasmic Reticulum Stress Contributes to Mitochondrial Exhaustion of CD8(+) T Cells. Cancer immunology research 2019;7(3):476–486. 30659052 10.1158/2326-6066.CIR-18-0182 PMC6397687

[130] Y. Cao , J. Trillo‐Tinoco , R. A. Sierra , et al. ER Stress‐induced Mediator C/EBP Homologous Protein Thwarts Effector T Cell Activity in Tumors Through T‐bet Repression. Nature Communications 2019;10(1):1280. 10.1038/s41467-019-09263-1 PMC6426975 30894532

[131] X. Li , J. Zheng , S. Chen , F. D. Meng , J. Ning , and S. L Sun . Oleandrin, a Cardiac Glycoside, Induces Immunogenic Cell Death via the PERK/elF2α/ATF4/CHOP Pathway in Breast Cancer. Cell death & disease 2021;12(4):314. 33762577 10.1038/s41419-021-03605-y PMC7990929

[132] M. Song , T. A. Sandoval , C. S. Chae , et al. IRE1α‐XBP1 controls T Cell Function in Ovarian Cancer by Regulating Mitochondrial Activity. Nature 2018;562(7727):423–428. 30305738 10.1038/s41586-018-0597-x PMC6237282

[133] X. Ma , E. Bi , Y. Lu , et al. Cholesterol Induces CD8(+) T Cell Exhaustion in the Tumor Microenvironment. Cell metabolism 2019;30(1):143–156.e5.e5. 31031094 10.1016/j.cmet.2019.04.002 PMC7061417

[134] Y. Chen , S. Zhang , Q. Wang , and X Zhang . Tumor‐recruited M2 Macrophages Promote Gastric and Breast Cancer Metastasis via M2 Macrophage‐secreted CHI3L1 Protein. Journal of hematology & oncology 2017;10(1):36. 28143526 10.1186/s13045-017-0408-0 PMC5286803

[135] M. J. Kim , H. J. Sun , Y. S. Song , et al. CXCL16 positively Correlated With M2‐macrophage Infiltration, Enhanced Angiogenesis, and Poor Prognosis in Thyroid Cancer. Scientific Reports 2019;9(1):13288. 31527616 10.1038/s41598-019-49613-z PMC6746802

[136] C. Wei , C. Yang , S. Wang , et al. Crosstalk Between Cancer Cells and Tumor Associated Macrophages Is Required for Mesenchymal Circulating Tumor Cell‐mediated Colorectal Cancer Metastasis. Molecular cancer 2019;18(1):64. 30927925 10.1186/s12943-019-0976-4 PMC6441214

[137] R. Wang , Y. Liu , L. Liu , et al. Tumor Cells Induce LAMP2a Expression in Tumor‐associated Macrophage for Cancer Progression. EBioMedicine 2019;40:118–134. 30711520 10.1016/j.ebiom.2019.01.045 PMC6413476

[138] B. Z. Qian and J. W Pollard . Macrophage Diversity Enhances Tumor Progression and Metastasis. Cell 2010;141(1):39–51. 20371344 10.1016/j.cell.2010.03.014 PMC4994190

[139] L. Qi , J. Chen , Y. Yang , and W Hu . Hypoxia Correlates with Poor Survival and M2 Macrophage Infiltration in Colorectal Cancer. Frontiers in oncology 2020;10:566430. 33330037 10.3389/fonc.2020.566430 PMC7714992

[140] D. Laoui , E. Van Overmeire , G. Di Conza , et al. Tumor Hypoxia Does Not Drive Differentiation of Tumor‐associated Macrophages but Rather Fine‐tunes the M2‐Like Macrophage Population. Cancer Research 2014;74(1):24–30. 24220244 10.1158/0008-5472.CAN-13-1196

[141] L. N. Raines , H. Zhao , Y. Wang , et al. PERK Is a Critical Metabolic Hub for Immunosuppressive Function in Macrophages. Nature Immunology 2022;23(3):431–445. 35228694 10.1038/s41590-022-01145-x PMC9112288

[142] F. Yang , Y. Liu , H. Ren , G. Zhou , X. Yuan , and X Shi . ER‐stress Regulates Macrophage Polarization Through Pancreatic EIF‐2alpha Kinase. Cellular Immunology 2019;336:40–47. 30594305 10.1016/j.cellimm.2018.12.008

[143] G. Di Conza , C. H. Tsai , H. Gallart‐Ayala , et al. Tumor‐induced Reshuffling of Lipid Composition on the Endoplasmic Reticulum Membrane Sustains Macrophage Survival and Pro‐tumorigenic Activity. Nature Immunology 2021;22(11):1403–1415. 34686867 10.1038/s41590-021-01047-4 PMC7611917

[144] M. Jiang , X. Li , J. Zhang , et al. Dual Inhibition of Endoplasmic Reticulum Stress and Oxidation Stress Manipulates the Polarization of Macrophages Under Hypoxia to Sensitize Immunotherapy. ACS Nano 2021;15(9):14522–14534. 34414762 10.1021/acsnano.1c04068

[145] M. S. Gilardini Montani , L. Falcinelli , and R. Santarelli , et al. KSHV Infection Skews Macrophage Polarisation towards M2‐Like/TAM and Activates Ire1 α‐XBP1 Axis Up‐regulating Pro‐tumorigenic Cytokine Release and PD‐L1 Expression. British Journal of Cancer 2020;123(2):298–306. 32418990 10.1038/s41416-020-0872-0 PMC7374093

[146] Y. Zhao , W. Zhang , M. Huo , et al. XBP1 regulates the Protumoral Function of Tumor‐associated Macrophages in human Colorectal Cancer. Signal Transduct Target Ther 2021;6(1):357. 34667145 10.1038/s41392-021-00761-7 PMC8526672

[147] H. Zhang , S. Q. Wang , L. Hang , et al. GRP78 facilitates M2 Macrophage Polarization and Tumour Progression. Cellular and Molecular Life Sciences 2021;78(23):7709–7732. 34713304 10.1007/s00018-021-03997-2 PMC11072571

[148] J. Liu , L. Fan , H. Yu , et al. Endoplasmic Reticulum Stress Causes Liver Cancer Cells to Release Exosomal miR‐23a‐3p and Up‐regulate Programmed Death Ligand 1 Expression in Macrophages. Hepatology 2019;70(1):241–258. 30854665 10.1002/hep.30607 PMC6597282

[149] X. Yao , Y. Tu , Y. Xu , Y. Guo , F. Yao , and X Zhang . Endoplasmic Reticulum Stress‐induced Exosomal miR‐27a‐3p Promotes Immune Escape in Breast Cancer via Regulating PD‐L1 Expression in Macrophages. Journal of Cellular and Molecular Medicine 2020;24(17):9560–9573. 32672418 10.1111/jcmm.15367 PMC7520328

[150] Y. Yuan , P. Jiao , Z. Wang , et al. Endoplasmic Reticulum Stress Promotes the Release of Exosomal PD‐L1 From Head and Neck Cancer Cells and Facilitates M2 Macrophage Polarization. Cell Communication and Signaling 2022;20(1):12. 35090495 10.1186/s12964-021-00810-2 PMC8796490

[151] N. R. Mahadevan , J. Rodvold , H. Sepulveda , S. Rossi , A. F. Drew , and M Zanetti . Transmission of Endoplasmic Reticulum Stress and Pro‐inflammation From Tumor Cells to Myeloid Cells. PNAS 2011;108(16):6561–6566. 21464300 10.1073/pnas.1008942108 PMC3081038

[152] V. Kumar , S. Patel , E. Tcyganov , and D. I Gabrilovich . The Nature of Myeloid‐Derived Suppressor Cells in the Tumor Microenvironment. Trends in Immunology 2016;37(3):208–220. 26858199 10.1016/j.it.2016.01.004 PMC4775398

[153] P. L. Raber , P. Thevenot , R. Sierra , et al. Subpopulations of Myeloid‐derived Suppressor Cells Impair T Cell Responses Through Independent Nitric Oxide‐related Pathways. International Journal of Cancer 2014;134(12):2853–2864. 24259296 10.1002/ijc.28622 PMC3980009

[154] J. M. Ju , G. Nam , Y. K. Lee , et al. IDO1 scavenges Reactive Oxygen Species in Myeloid‐derived Suppressor Cells to Prevent Graft‐versus‐host Disease. PNAS 2021;118(10). 10.1073/pnas.2011170118 PMC7958359 33649207

[155] K. Cole , K. Pravoverov , and J. E Talmadge . Role of Myeloid‐derived Suppressor Cells in Metastasis. Cancer and Metastasis Reviews 2021;40(2):391–411. 33411082 10.1007/s10555-020-09947-x

[156] N. Erin , J. Grahovac , A. Brozovic , and T Efferth . Tumor Microenvironment and Epithelial Mesenchymal Transition as Targets to Overcome Tumor Multidrug Resistance. Drug Resistance Updates 2020;53:100715. 32679188 10.1016/j.drup.2020.100715

[157] P. De Cicco , G. Ercolano , and A Ianaro . The New Era of Cancer Immunotherapy: Targeting Myeloid‐Derived Suppressor Cells to Overcome Immune Evasion. Frontiers in immunology 2020;11:1680. 32849585 10.3389/fimmu.2020.01680 PMC7406792

[158] E. N. Tcyganov , S. Hanabuchi , A. Hashimoto , et al. Distinct Mechanisms Govern Populations of Myeloid‐derived Suppressor Cells in Chronic Viral Infection and Cancer. Journal of Clinical Investigation 2021;131(16). 10.1172/JCI145971 PMC8363287 34228641

[159] T. Condamine , G. A. Dominguez , J. I. Youn , et al. Lectin‐type Oxidized LDL Receptor‐1 Distinguishes Population of human Polymorphonuclear Myeloid‐derived Suppressor Cells in Cancer Patients. Science Immunology 2016;1(2). 10.1126/sciimmunol.aaf8943 PMC5391495 28417112

[160] E. Mohamed , R. A. Sierra , and J. Trillo‐Tinoco , et al. The Unfolded Protein Response Mediator PERK Governs Myeloid Cell‐Driven Immunosuppression in Tumors Through Inhibition of STING Signaling. Immunity 2020;52(4):668–682.e7. 32294407 10.1016/j.immuni.2020.03.004 PMC7207019

[161] R. A. Sierra , J. Trillo‐Tinoco , E. Mohamed , et al. Anti‐Jagged Immunotherapy Inhibits MDSCs and Overcomes Tumor‐Induced Tolerance. Cancer Research 2017;77(20):5628–5638. 28904063 10.1158/0008-5472.CAN-17-0357 PMC5679354

[162] P. T. Thevenot , R. A. Sierra , P. L. Raber , et al. The Stress‐response Sensor Chop Regulates the Function and Accumulation of Myeloid‐derived Suppressor Cells in Tumors. Immunity 2014;41(3):389–401. 25238096 10.1016/j.immuni.2014.08.015 PMC4171711

[163] M. Liu , C. Wu , S. Luo , et al. PERK Reprograms Hematopoietic Progenitor Cells to Direct Tumor‐promoting Myelopoiesis in the Spleen. Journal of Experimental Medicine 2022;219(4). 10.1084/jem.20211498 PMC8919616 35266960

[164] L. B. Kennedy and A. K. S Salama . A Review of Cancer Immunotherapy Toxicity. CA: A Cancer Journal for Clinicians 2020;70(2):86–104. 31944278 10.3322/caac.21596

[165] F. Conforti , L. Pala , V. Bagnardi , et al. Cancer Immunotherapy Efficacy and Patients' sex: A Systematic Review and Meta‐analysis. The Lancet Oncology 2018;19(6):737–746. 29778737 10.1016/S1470-2045(18)30261-4

[166] M. Aldea , F. Andre , A. Marabelle , S. Dogan , F. Barlesi , and J. C Soria . Overcoming Resistance to Tumor‐Targeted and Immune‐Targeted Therapies. Cancer discovery 2021;11(4):874–899. 33811122 10.1158/2159-8290.CD-20-1638

[167] M. A. Arap , J. Lahdenranta , P. J. Mintz , et al. Cell Surface Expression of the Stress Response Chaperone GRP78 Enables Tumor Targeting by Circulating Ligands. Cancer Cell 2004;6(3):275–284. 15380518 10.1016/j.ccr.2004.08.018

[168] C. Kao , R. Chandna , A. Ghode , et al. Proapoptotic Cyclic Peptide BC71 Targets Cell‐Surface GRP78 and Functions as an Anticancer Therapeutic in Mice. EBioMedicine 2018;33:22–32. 29907328 10.1016/j.ebiom.2018.06.004 PMC6085501

[169] M. Chen , Y. Zhang , V. C. Yu , Y. S. Chong , T. Yoshioka , and R Ge . Isthmin Targets Cell‐surface GRP78 and Triggers Apoptosis via Induction of Mitochondrial Dysfunction. Cell Death and Differentiation 2014;21(5):797–810. 24464222 10.1038/cdd.2014.3 PMC3978310

[170] R. Burikhanov , Y. Zhao , A. Goswami , S. Qiu , S. R. Schwarze , and V. M Rangnekar . The Tumor Suppressor Par‐4 Activates an Extrinsic Pathway for Apoptosis. Cell 2009;138(2):377–388. 19632185 10.1016/j.cell.2009.05.022 PMC2774252

[171] A. W. Paton , T. Beddoe , C. M. Thorpe , et al. AB5 subtilase Cytotoxin Inactivates the Endoplasmic Reticulum Chaperone BiP. Nature 2006;443(7111):548–552. 17024087 10.1038/nature05124

[172] S. Samanta , S. Yang , B. Debnath , et al. The Hydroxyquinoline Analogue YUM70 Inhibits GRP78 to Induce ER Stress‐Mediated Apoptosis in Pancreatic Cancer. Cancer Research 2021;81(7):1883–1895. 33531374 10.1158/0008-5472.CAN-20-1540 PMC8137563

[173] Y. Qiao , C. Dsouza , A. A. Matthews , et al. Discovery of Small Molecules Targeting GRP78 for Antiangiogenic and Anticancer Therapy. European Journal of Medicinal Chemistry 2020;193:112228. 32199134 10.1016/j.ejmech.2020.112228

[174] N. Hebbar , R. Epperly , A. Vaidya , et al. CAR T Cells Redirected to Cell Surface GRP78 Display Robust Anti‐acute Myeloid Leukemia Activity and Do Not Target Hematopoietic Progenitor Cells. Nature Communications 2022;13(1):587. 10.1038/s41467-022-28243-6 PMC8803836 35102167

[175] A. V. Korennykh , P. F. Egea , A. A. Korostelev , et al. The Unfolded Protein Response Signals Through High‐order Assembly of Ire1. Nature 2009;457(7230):687–693. 19079236 10.1038/nature07661 PMC2846394

[176] L. Wang , B. G. Perera , S. B. Hari , et al. Divergent Allosteric Control of the IRE1α Endoribonuclease Using Kinase Inhibitors. Nature Chemical Biology 2012;8(12):982–989. 23086298 10.1038/nchembio.1094 PMC3508346

[177] R. Ghosh , L. Wang , E. S. Wang , et al. Allosteric Inhibition of the IRE1α RNase Preserves Cell Viability and Function During Endoplasmic Reticulum Stress. Cell 2014;158(3):534–548. 25018104 10.1016/j.cell.2014.07.002 PMC4244221

[178] M. Thamsen , R. Ghosh , V. C. Auyeung , et al. Small Molecule Inhibition of IRE1α Kinase/RNase Has Anti‐fibrotic Effects in the Lung. PLoS ONE 2019;14(1):e0209824. 30625178 10.1371/journal.pone.0209824 PMC6326459

[179] S. Morita , S. A. Villalta , H. C. Feldman , et al. Targeting ABL‐IRE1α Signaling Spares ER‐Stressed Pancreatic β Cells to Reverse Autoimmune Diabetes. Cell metabolism 2017;25(4):883–897.e8.e8. 28380378 10.1016/j.cmet.2017.03.018 PMC5497784

[180] H. C. Feldman , R. Ghosh , V. C. Auyeung , et al. ATP‐competitive Partial Antagonists of the IRE1α RNase Segregate Outputs of the UPR. Nature Chemical Biology 2021;17(11):1148–1156. 34556859 10.1038/s41589-021-00852-0 PMC8551014

[181] B. C. Cross , P. J. Bond , P. G. Sadowski , et al. The Molecular Basis for Selective Inhibition of Unconventional mRNA Splicing by an IRE1‐binding Small Molecule. PNAS 2012;109(15):E869–E878. 22315414 10.1073/pnas.1115623109 PMC3326519

[182] I. Papandreou , N. C. Denko , M. Olson , et al. Identification of an Ire1alpha Endonuclease Specific Inhibitor With Cytotoxic Activity Against human Multiple Myeloma. Blood 2011;117(4):1311–1314. 21081713 10.1182/blood-2010-08-303099 PMC3056474

[183] N. McCarthy , N. Dolgikh , S. Logue , et al. The IRE1 and PERK Arms of the Unfolded Protein Response Promote Survival of Rhabdomyosarcoma Cells. Cancer Letters 2020;490:76–88. 32679165 10.1016/j.canlet.2020.07.009

[184] C. Atkins , Q. Liu , E. Minthorn , et al. Characterization of a Novel PERK Kinase Inhibitor With Antitumor and Antiangiogenic Activity. Cancer Research 2013;73(6):1993–2002. 23333938 10.1158/0008-5472.CAN-12-3109

[185] J. M. Axten , J. R. Medina , Y. Feng , et al. Discovery of 7‐methyl‐5‐(1‐{[3‐(trifluoromethyl)phenyl]Acetyl}‐2,3‐dihydro‐1H‐indol‐5‐yl)‐7H‐pyrrolo[2,3‐d]Pyrimidin‐4‐amine (GSK2606414), a Potent and Selective First‐in‐class Inhibitor of Protein Kinase R (PKR)‐Like Endoplasmic Reticulum Kinase (PERK). Journal of Medicinal Chemistry 2012;55(16):7193–7207. 22827572 10.1021/jm300713s

[186] C. M. Gallagher , C. Garri , and E. L. Cain , et al. Ceapins Are a New Class of Unfolded Protein Response Inhibitors, Selectively Targeting the ATF6α Branch. Elife 2016;5. 10.7554/eLife.11878 PMC4954757 27435960

[187] L. J. Bu , H. Q. Yu , L. L. Fan , et al. Melatonin, a Novel Selective ATF‐6 Inhibitor, Induces human Hepatoma Cell Apoptosis Through COX‐2 Downregulation. World Journal of Gastroenterology 2017;23(6):986. 28246472 10.3748/wjg.v23.i6.986 PMC5311108

[188] A. S Lee . Glucose‐regulated Proteins in Cancer: Molecular Mechanisms and Therapeutic Potential. Nature Reviews Cancer 2014;14(4):263–276. 24658275 10.1038/nrc3701 PMC4158750

[189] D. I. Staquicini , S. D'Angelo , and F. Ferrara , et al. Therapeutic Targeting of Membrane‐associated GRP78 in Leukemia and Lymphoma: Preclinical Efficacy in Vitro and Formal Toxicity Study of BMTP‐78 in Rodents and Primates. Pharmacogenomics Journal 2018;18(3):436–443. 29205207 10.1038/tpj.2017.46 PMC6824482

[190] Y. R. Miao , B. L. Eckhardt , Y. Cao , et al. Inhibition of Established Micrometastases by Targeted Drug Delivery via Cell Surface‐associated GRP78. Clinical Cancer Research 2013;19(8):2107–2116. 23470966 10.1158/1078-0432.CCR-12-2991 PMC4331071

[191] J. Ibanez , N. Hebbar , U. Thanekar , et al. GRP78‐CAR T Cell Effector Function Against Solid and Brain Tumors Is Controlled by GRP78 Expression on T Cells. Cell Rep Med 2023;4(11):101297. 37992682 10.1016/j.xcrm.2023.101297 PMC10694756

[192] D. P. Raymundo , D. Doultsinos , X. Guillory , A. Carlesso , L. A. Eriksson , and E Chevet . Pharmacological Targeting of IRE1 in Cancer. Trends in cancer 2020;6(12):1018–1030. 32861679 10.1016/j.trecan.2020.07.006

[193] M. Maurel , E. Chevet , J. Tavernier , and S Gerlo . Getting RIDD of RNA: IRE1 in Cell Fate Regulation. Trends in Biochemical Sciences 2014;39(5):245–254. 24657016 10.1016/j.tibs.2014.02.008

[194] A. Carlesso , C. Chintha , A. M. Gorman , A. Samali , and L. A Eriksson . Effect of Kinase Inhibiting RNase Attenuator (KIRA) Compounds on the Formation of Face‐to‐Face Dimers of Inositol‐Requiring Enzyme 1: Insights From Computational Modeling. International Journal of Molecular Sciences 5538, 2019;20(22). 31698846 10.3390/ijms20225538 PMC6887741

[195] J. M. Harnoss , A. Le Thomas , A. Shemorry , et al. Disruption of IRE1α Through Its Kinase Domain Attenuates Multiple Myeloma. PNAS 2019;116(33):16420–16429. 31371506 10.1073/pnas.1906999116 PMC6697881

[196] S. E. Logue , E. P. McGrath , P. Cleary , et al. Inhibition of IRE1 RNase Activity Modulates the Tumor Cell Secretome and Enhances Response to Chemotherapy. Nature Communications 2018;9(1):3267. 10.1038/s41467-018-05763-8 PMC6093931 30111846

[197] X. Sheng , H. Z. Nenseth , S. Qu , et al. IRE1α‐XBP1s pathway Promotes Prostate Cancer by Activating c‐MYC Signaling. Nature Communications 2019;10(1):323. 10.1038/s41467-018-08152-3 PMC6345973 30679434

[198] R. Xiao , L. You , L. Zhang , et al. Inhibiting the IRE1α Axis of the Unfolded Protein Response Enhances the Antitumor Effect of AZD1775 in TP53 Mutant Ovarian Cancer. Adv Sci (Weinh) 2022;9(21):e2105469. 35619328 10.1002/advs.202105469 PMC9313493

[199] P. J. Le Reste , R. Pineau , and K. Voutetakis , et al. Local Intracerebral Inhibition of IRE1 by MKC8866 Sensitizes Glioblastoma to Irradiation/Chemotherapy in Vivo. Cancer Letters 2020;494:73–83. 32882336 10.1016/j.canlet.2020.08.028

[200] D. Rojas‐Rivera , T. Delvaeye , R. Roelandt , et al. When PERK Inhibitors Turn out to be New Potent RIPK1 Inhibitors: Critical Issues on the Specificity and Use of GSK2606414 and GSK2656157. Cell Death and Differentiation 2017;24(6):1100–1110. 28452996 10.1038/cdd.2017.58 PMC5442476

[201] Z. Li , Y. Ge , J. Dong , et al. BZW1 Facilitates Glycolysis and Promotes Tumor Growth in Pancreatic Ductal Adenocarcinoma through Potentiating eIF2α Phosphorylation. Gastroenterology 2022;162(4):1256–1271.e14.e14. 34951995 10.1053/j.gastro.2021.12.249 PMC9436032

[202] W. Cai , X. Sun , and F. Jin , et al. PERK‐eIF2α‐ERK1/2 Axis Drives Mesenchymal‐endothelial Transition of Cancer‐associated Fibroblasts in Pancreatic Cancer. Cancer Letters 2021;515:86–95. 34052329 10.1016/j.canlet.2021.05.021

[203] T. Bagratuni , D. Patseas , N. Mavrianou‐Koutsoukou , et al. Characterization of a PERK Kinase Inhibitor With Anti‐Myeloma Activity. Cancers (Basel) 2864, 2020;12(10). 33028016 10.3390/cancers12102864 PMC7601861

[204] Y. Gao , D. J. Sartori , C. Li , et al. PERK Is Required in the Adult Pancreas and Is Essential for Maintenance of Glucose Homeostasis. Molecular and Cellular Biology 2012;32(24):5129–5139. 23071091 10.1128/MCB.01009-12 PMC3510549

[205] O. I. Coleman , E. M. Lobner , S. Bierwirth , et al. Activated ATF6 Induces Intestinal Dysbiosis and Innate Immune Response to Promote Colorectal Tumorigenesis. Gastroenterology 2018;155(5):1539–1552.e12.e12. 30063920 10.1053/j.gastro.2018.07.028

[206] M. Shuda , N. Kondoh , N. Imazeki , et al. Activation of the ATF6, XBP1 and grp78 Genes in human Hepatocellular Carcinoma: A Possible Involvement of the ER Stress Pathway in Hepatocarcinogenesis. Journal of Hepatology 2003;38(5):605–614. 12713871 10.1016/s0168-8278(03)00029-1

[207] J. Cho , H. Y. Min , H. Pei , et al. The ATF6‐EGF Pathway Mediates the Awakening of Slow‐Cycling Chemoresistant Cells and Tumor Recurrence by Stimulating Tumor Angiogenesis. Cancers (Basel) 1772, 2020;12(7). 32630838 10.3390/cancers12071772 PMC7407555

[208] B. Farhood , N. H. Goradel , K. Mortezaee , et al. Melatonin as an Adjuvant in Radiotherapy for Radioprotection and Radiosensitization. Clinical & translational oncology 2019;21(3):268–279. 30136132 10.1007/s12094-018-1934-0

[209] Y. Y. Shin , Y. Seo , S. J. Oh , et al. Melatonin and Verteporfin Synergistically Suppress the Growth and Stemness of Head and Neck Squamous Cell Carcinoma Through the Regulation of Mitochondrial Dynamics. Journal of Pineal Research 2022;72(1):e12779. 34826168 10.1111/jpi.12779

[210] J. Wu , Z. Tan , H. Li , et al. Melatonin Reduces Proliferation and Promotes Apoptosis of Bladder Cancer Cells by Suppressing O‐GlcNAcylation of Cyclin‐dependent‐Like Kinase 5. Journal of Pineal Research 2021;71(3):e12765. 34487576 10.1111/jpi.12765

[211] N. Chignard , S. Shang , H. Wang , et al. Cleavage of Endoplasmic Reticulum Proteins in Hepatocellular Carcinoma: Detection of Generated Fragments in Patient Sera. Gastroenterology 2006;130(7):2010–2022. 16762624 10.1053/j.gastro.2006.02.058

[212] Z. Su , L. Wang , X. Chen , et al. An Unfolded Protein Response‐Related mRNA Signature Predicting the Survival and Therapeutic Effect of Hepatocellular Carcinoma. Combinatorial Chemistry & High Throughput Screening 2022;25(12):2046–2058. 35125080 10.2174/1386207325666220204140925

[213] H. Guo , S. Zhang , B. Zhang , et al. Immunogenic Landscape and Risk Score Prediction Based On Unfolded Protein Response (UPR)‐related Molecular Subtypes in Hepatocellular Carcinoma. Frontiers in immunology 2023;14:1202324. 37457742 10.3389/fimmu.2023.1202324 PMC10348016

[214] A. Houessinon , A. Gicquel , F. Bochereau , et al. Alpha‐fetoprotein Is a Biomarker of Unfolded Protein Response and Altered Proteostasis in Hepatocellular Carcinoma Cells Exposed to Sorafenib. Cancer Letters 2016;370(2):242–249. 26546044 10.1016/j.canlet.2015.10.032

[215] A. Galmiche , C. Sauzay , A. Houessinon , B. Chauffert , and O Pluquet . Probing Tumour Proteostasis and the UPR With Serum Markers. Trends in cancer 2016;2(5):219–221. 28741509 10.1016/j.trecan.2016.04.004

[216] J. Tang , Y. S. Guo , Y. Zhang , et al. CD147 induces UPR to Inhibit Apoptosis and Chemosensitivity by Increasing the Transcription of Bip in Hepatocellular Carcinoma. Cell Death and Differentiation 2012;19(11):1779–1790. 22595757 10.1038/cdd.2012.60 PMC3469060

[217] L. Rasche , J. Duell , I. C. Castro , et al. GRP78‐directed Immunotherapy in Relapsed or Refractory Multiple Myeloma—results From a Phase 1 Trial With the Monoclonal Immunoglobulin M Antibody PAT‐SM6. Haematologica 2015;100(3):377–384. 25637055 10.3324/haematol.2014.117945 PMC4349277

[218] F. Hong , C. Y. Lin , J. Yan , et al. Canopy Homolog 2 Contributes to Liver Oncogenesis by Promoting Unfolded Protein Response‐dependent Destabilization of Tumor Protein P53. Hepatology 2022;76(6):1587–1601. 34986508 10.1002/hep.32318

[219] A. D'Urso , F. Oltolina , C. Borsotti , M. Prat , D. Colangelo , and A Follenzi . Macrophage Reprogramming via the Modulation of Unfolded Protein Response With siRNA‐Loaded Magnetic Nanoparticles in a TAM‐Like Experimental Model. Pharmaceutics 2023;15(6). 10.3390/pharmaceutics15061711 PMC10302170 37376159

[220] S. Rahman , V. Kumar , A. Kumar , T. S. Abdullah , I. A. Rather , and A. T Jan . Molecular Perspective of Nanoparticle Mediated Therapeutic Targeting in Breast Cancer: An Odyssey of Endoplasmic Reticulum Unfolded Protein Response (UPR(ER)) and beyond. Biomedicines 635, 2021;9(6). 34199484 10.3390/biomedicines9060635 PMC8229605

[221] J. Wang , X. Fang , and W Liang . Pegylated Phospholipid Micelles Induce Endoplasmic Reticulum‐dependent Apoptosis of Cancer Cells but Not Normal Cells. ACS Nano 2012;6(6):5018–5030. 22578158 10.1021/nn300571c

[222] Z. Gao , B. Jing , Y. Wang , W. Wan , X. Dong , and Y Liu . Exploring the Impact of Lipid Nanoparticles on Protein Stability and Cellular Proteostasis. Journal of Colloid & Interface Science 2025;678(Pt A):656–665. 39216393 10.1016/j.jcis.2024.08.146

